# Pro-angiogenic New Chloro-Azaphilone Derivatives From the Hadal Trench-Derived Fungus *Chaetomium globosum* YP-106

**DOI:** 10.3389/fmicb.2022.943452

**Published:** 2022-07-22

**Authors:** Yaqin Fan, Chunjiao Jiang, Yan Zhang, Zhiheng Ma, Peihai Li, Lizhong Guo, Ting Feng, Liman Zhou, Lili Xu

**Affiliations:** ^1^Shandong Provincial Key Laboratory of Applied Mycology, School of Life Sciences, Qingdao Agricultural University, Qingdao, China; ^2^Shandong Provincial Engineering Laboratory for Biological Testing Technology, Key Laboratory for Biosensor of Shandong Province, Engineering Research Center of Zebrafish Models for Human Diseases and Drug Screening of Shandong Province, Biology Institute, Qilu University of Technology (Shandong Academy of Sciences), Jinan, China; ^3^Key Laboratory of Chemistry and Engineering of Forest Products, State Ethnic Affairs Commission, Guangxi Key Laboratory of Chemistry and Engineering of Forest Products, Guangxi Collaborative Innovation Center for Chemistry and Engineering of Forest Products, School of Chemistry and Chemical Engineering, Guangxi Minzu University, Nanning, China

**Keywords:** hadal trench-derived fungus, metabolites, chloro-azaphilones, pro-angiogenic activity, *Chaetomium globosum*

## Abstract

Five new chloro-azaphilones, chaetofanixins A–E (1–5), and five known analogs (6–10) were isolated and identified from the hadal trench-derived fungus *Chaetomium globosum* YP-106. The structure of chaetofanixin E (5) is unique and interesting, bearing a highly rigid 6/6/5/3/5 penta-cyclic ring system, which is first encountered in natural products. The structures of these compounds, including absolute configurations, were determined based on the spectroscopic analysis, electronic circular dichroism (ECD) calculations, and analysis of biogenetic origins. Compounds 1–7 significantly promoted angiogenesis in a dose-dependent manner, and thus, these compounds might be used as promising molecules for the development of natural cardiovascular disease agents.

## Introduction

The prevention and treatment of cardiovascular diseases (CVDs) is an important project in the field of medicine and food. CVD has emerged as one of the major diseases endangering human health, causing more than 17 million deaths worldwide every year (Virani et al., 2020). Insufficient angiogenesis is one of the most common causes of CVD, accompanied by symptoms, such as myocardial ischemia, cerebral ischemia, vasculitis obliterans, peripheral vascular disease, etc. (Jude et al., 2010; Kurusamy et al., 2017). In recent years, pro-angiogenesis has been highly valued as a new target for the development of cardiovascular drugs, and various therapeutic angiogenesis studies have been carried out in animal models and clinical practice (Li et al., 2021; Zhou et al., 2021). Clinical therapeutic angiogenesis approaches are diverse, such as cytokine therapy, endothelial progenitor cell therapy, stem cell therapy, gene therapy, and mechanical therapy. However, the above-mentioned methods have shortcomings, including transient efficacy, strong toxic side effects, and low success rate (Potz et al., 2017). Food prevention and drug treatment are important methods to prevent and treat cardiovascular diseases. The extraction of CVD drugs from microbial natural products has been considered an effective strategy for the prevention and treatment of CVDs. In the past 50 years, about 20,000 natural products have been reported from marine microorganisms, but the number of natural products reported from deep-sea microorganisms is very low, less than 2%, and hadal trench (>6,000 m) microorganisms are hardly reported (Skropeta and Wei, 2014; Carroll et al., 2021). In recent years, with the rapid development of sample collection, identification, and culture techniques of microorganisms, the chemical study of deep-sea fungi has shown a dramatic increase, and researchers have found a wealth of novel active secondary metabolites with pro-angiogenic (Fan et al., 2015; Yan et al., 2022), antibacterial (Wang et al., 2016; Chi et al., 2020b), anti-inflammatory (Guo et al., 2021), anticancer (Chi et al., 2020a), and other biological activities (Wang et al., 2020) from deep-sea microorganisms.

**FIGURE 1:**
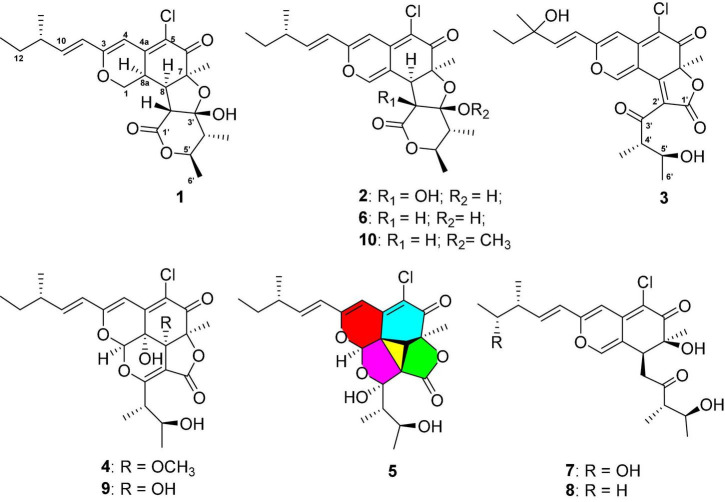
Structures of compounds 1–10.

In our continuous efforts for discovering structurally novel bioactive natural compounds from marine-derived microorganisms inhabiting unique environments, a fungal strain YP-106, identified as *Chaetomium globosum*, was isolated from the hadal zone seawater collected at a depth of 6,215 m from Yap Trench in the western Pacific Ocean. A subsequent chemical investigation of this fungus led to the identification of five new chloro*-*azaphilone derivatives, chaetofanixins A–E (1–5), and five related known azaphilones, chaetomugilin D (6) (Qin et al., 2009), chaetomugilin Q (7) (Yamada et al., 2011), chaetoviridin J (8) (Youn et al., 2015), chaetoviridin I (9) (Borges et al., 2011), and chaetomugilin E (10) (Youn et al., 2015) (Figure 1). Chaetofanixin E (5) is a novel chloro-azaphilone bearing a unique 6/6/5/3/5 penta-cyclic ring system. Compounds 1-7 exhibited the noteworthy pro-angiogenic effect in a dose-dependent manner. Herein, the isolation, structure identification, and bioactivity of these compounds are reported.

## Materials and Methods

### General Experimental Procedures

The ^1^H, ^13^C NMR, DEPT, and 2D NMR spectra were recorded on a Bruker Avance 600 spectrometer (Bruker). HRESIMS were measured on a Q-TOF Ultima Global GAA076 LC mass spectrometer. UV spectra were recorded on a 2487 detector of Waters. Electronic circular dichroism (ECD) spectra were measured on a JASCO J-815 spectrometer. Optical rotations were recorded with a JASCO P-1020 digital polarimeter. Sephadex LH-20 (Pharmacia Biotec AB, Uppsala, Sweden) and reverse-phase C18 silica gel (Merck, Darmstadt, Germany) were used for column chromatography (CC). Thin-layer chromatography (TLC) was performed on plates precoated with silica gel GF 254 (10–40 μm) and silica gel (200–300 mesh) (Qingdao Haiyang Chemical Co., Qingdao, China). Vacuum-liquid chromatography (VLC) used silica gel H (Qingdao Marine Chemical Factory). All solvents used were of analytical grade (Sinopharm Chemical Reagent Co., Shanghai, China). Semipreparative HPLC was performed using an ODS column (YMC-pack ODS-A, 10 × 250 mm, 5 μm, 4.0 mL/min).

### Collection and Phylogenetic Analysis of Strain YP-106

The fungus YP-106 was originally obtained from a seawater sample collected from the Yap Trench of the Pacific Ocean at a depth of 6,215 m in 2017. The sample (2 g) was diluted to 10^–3^ g/mL, and 100 μL of this solution was dispersed across a solid-phase agar plate (potato dextrose agar/PDA agar media) and incubated at 28°C for 4 days. A single colony was transferred to PDA agar media. The fungus was identified using a molecular biological protocol by DNA amplification and sequencing of the ITS and β-tubulin regions (Wang et al., 2006), as both showed a 99% identity to *Chaetomium globosum*. The sequence is deposited in GenBank under accession no. OL872214.

### Fermentation, Extraction, and Isolation

The fungus *Chaetomium globosum* YP-106 was aseptically transferred and grown under static conditions at 28°C for 30 days in fifty 1,000 mL conical flasks containing liquid nutrition medium (300 mL/flask) composed of malt extract (17 g/L), glucose (3 g/L), peptone (3 g/L), and sea salt (33 g/L), and tap water was added later. After the completion of the fermentation period, the whole fermentation broth (60 L) was filtered, and the broth media was extracted with ethyl acetate (EtOAc), while the solid mycelia were extracted by 85% (v/v) aqueous acetone. The combined extracts (105 g) of the fermentation broth and mycelia were eluted with a stepwise gradient of petroleum ether (PE):ethyl acetate (EtOAc) (80:1, 50:1, 20:1, 10:1, 8:1, 4:1, 2:1, and 1:1, v/v, 2 L each) and chloroform (CHCl_3_) : methyl alcohol (MeOH) (50:1, 20:1, 10:1, 5:1, 1:1, and 0:1, v/v, 2 L each) and fractionated into six fractions (Fr.1-Fr.6) over a silica gel (200-300 mesh) vacuum-liquid chromatography (VLC) column. Fr. 2 (25.8 g) was further purified by column chromatography (CC) on Sephadex LH-20 (1:1, v/v, CHCl_3_:MeOH) to obtain nine subfractions (Fr.2.1–2.9). Subfraction 2.5 was further subjected to HPLC purification using octadecylsilyl (ODS) silica gel column (MeOH-H_2_O, 75:25, v/v) to yield compounds 6 (34.2 mg) and 10 (33.5 mg). Fraction 3 (36.8 g) was further separated into four subfractions by Sephadex LH-20 CC using 100% MeOH. Subfraction 3.2 was further purified using HPLC on ODS (MeCN-H_2_O, 35:65, v/v) to yield 4 (1.1 mg), 3 (2.1 mg), and Fr.3.2.3. Subfraction 3.2.3 was further purified using preparative thin-layer chromatography (TLC) (PE–EtOAc, 1:1) to get 5 (1.1 mg) and 1 (7.8 mg). Fr. 4 (28.1 g) was subjected to CC on Sephadex LH-20 by eluting with 100% MeOH to yield six subfractions (Fr. 4.1–4.6). Subfraction 4.3 was further purified using HPLC on ODS (MeCN-H_2_O, 45:55, v/v) to get 9 (4.3 mg) and Fr.4.3.2. Subfraction 4.3.2 was further purified using preparative TLC (PE–EtOAc, 1:1) to get 2 (5.3 mg) and 8 (9.5 mg). Fr.5 (9.8 g) was disposed of reverse-phase silica gel (ODS) using stepwise gradient elution with MeOH/H_2_O (10–100%) to obtain four subfractions (Fr.5.1–Fr.5.4). Subfraction 5.3 was further purified by Sephadex LH-20 CC using 100% MeOH and then followed by HPLC (MeCN-H_2_O, 35:65, v/v) to yield 7 (1.2 mg).

*Chaetofanixin A (1).* This is a yellow amorphous powder; [α]25 D –47 (*c* 0.1, CHCl_3_); UV (MeOH) λ_*max*_ 217 (4.58), 238 (4.82), 282 (4.26), and 373 (5.50) nm; CD (*c* 0.1, MeOH) λ_*max*_ (Δε) 205 (–3.14), 223 (+3.89), 242 (–1.87), 320 (+0.63), and 377 (–3.38); ^1^H and ^13^C NMR data, see Table 1; HRESIMS m/z 437.1729 [M+H]^+^ (calcd for C_23_H_30_O_6_Cl, 437.1725).

**TABLE 1:** NMR data of compounds 1–5 in DMSO-*d*_6_ (600 MHz for ^1^H and 150 MHz for ^13^C, δ in ppm, *J* in Hz).

No.	1		2		3		4		5	
	δ_*C*_	δ_*H*_	δ_*C*_	δ_*H*_	δ_*C*_	δ_*H*_	δ_*C*_	δ_*H*_	δ_*C*_	δ_*H*_
1	68.5, CH_2_	4.41 (1H, dd, 11.4, 5.0);	144.4, CH	7.25 (1H, s)	150.8, CH	8.62 (1H, s)	96.2, CH	6.00 (1H, s)	99.2, CH	5.74 (1H, s)
		4.15 (1H, dd, 13.6,11.4)								
3	161.7, C		156.0, C		157.2, C		155.8, C		160.5, C	
4	101.3, CH	6.11 (1H, s)	104.6, CH	6.54 (1H, s)	105.7, CH	6.88 (1H, s)	101.4, CH	6.20 (1H, s)	100.0, CH	6.09 (1H, s)
4a	144.5, C		141.7, C		140.2, C		144.1, C		141.0, C	
5	119.0, C		109.4, C		107.4, C		123.0, C		117.3, C	
6	188.9, C		188.1, C		183.2, C		186.2, C		183.9, C	
7	83.6, C		81.9, C		87.3, C		85.9, C		80.5, C	
8	48.9, CH	2.83 (1H, overlap)	52.4, CH	3.35 (1H, overlap)	160.5, C		77.2, C		39.1, CH	2.93 (1H, s)
8a	33.3, CH	3.39 (1H, ddd, 13.6, 5.0, 2.0)	112.0, C		110.0, C		65.3, C		39.2, C	
9	122.8, CH	6.19 (1H, d, 15.6)	120.9, CH	6.35 (1H, d, 15.8)	118.4, CH	6.54 (1H, d, 15.7)	123.0, CH	6.28 (1H, d, 15.2)	123.9, CH	6.19 (1H, d, 15.7)
10	144.8, CH	6.39 (1H, dd, 15.6, 7.8)	145.2, CH	6.43 (1H, dd, 15.8,7.6)	148.5, CH	6.74 (1H, d, 15.7)	146.4, CH	6.52 (1H, dd, 15.2, 6.7)	146.1, CH	6.39 (1H, dd, 15.7,7.9)
11	37.8 CH	2.23 (1H, m)	38.0, CH	2.25 (1H, m)	72.0, C		38.4, CH	2.27 (1H, m)	38.5, CH	2.22 (1H, m)
12	28.6, CH_2_	1.36 (2H, m)	28.6, CH_2_	1.39 (2H, m)	34.4, CH_2_	1.54 (1H, dd, 14.9, 7.4)	29.1, CH_2_	1.40 (2H, m)	29.2, CH_2_	1.37 (2H, m)
						1.53 (1H, dd, 14.9, 7.4)				
13	11.6, CH_3_	0.85 (3H, t,7.4)	11.6, CH_3_	0.85 (3H, t,7.4)	8.2, CH_3_	0.82 (3H, t, 7.4)	12.1, CH_3_	0.85 (3H, t,7.4)	12.3, CH_3_	0.85 (3H, t, 7.4)
7-Me	21.5, CH_3_	1.39 (3H, s)	24.2, CH_3_	1.26 (3H, s)	25.2, CH_3_	1.63 (3H, s)	18.3, CH_3_	1.61 (3H, s)	20.0, CH_3_	1.55 (3H, s)
11-Me	19.2, CH_3_	1.01 (3H, d, 6.7)	19.4, CH_3_	1.02 (3H, d,6.9)	27.3, CH_3_	1.23 (3H, s)	19.4, CH_3_	1.04 (3H, d, 6.7)	19.7, CH_3_	1.02 (3H, d, 6.8)
1′	171.4, C		170.7, C		168.0, C		166.7, C		167.7, C	
2′	54.3, CH	2.82 (1H, overlap)	81.7, C		125.9, C		98.3, C		55.0, C	
3′	104.2, C		104.6, C		200.9, C		169.6, C		107.8, C	
4′	45.3, CH	1.64 (1H, dq, 9.4, 6.9)	46.2, CH	1.65 (1H, dq, 9.2, 6.7)	50.5, CH	3.41 (1H, dq, 8.6, 6.3)	40.9, CH	3.38 (1H, dq, 10.9, 6.7)	47.9, CH	1.64(1H, dq, 9.2, 7.1)
5′	76.1, CH	4.27 (1H, dq, 9.4, 6.4)	75.3, CH	4.37 (1H, dq, 9.2, 6.2)	69.6, CH	3.56 (1H, m)	68.0, CH	3.40 (1H, dq, 10.9, 6.7)	66.3, CH	3.70 (1H, ddq, 9.2, 3.8, 6.6)
6′	18.3, CH_3_	1.24 (3H, d, 6.4)	18.5, CH_3_	1.27 (3H, d, 6.2)	21.5, CH_3_	0.98 (3H, d, 6.2)	20.9, CH_3_	0.92 (3H, d, 6.7)	22.1, CH_3_	1.03 (3H, d, 6.6)
4′-Me	8.8, CH_3_	0.95 (3H, d, 6.9)	9.5, CH_3_	1.01 (3H, d, 6.7)	12.7, CH_3_	0.99 (3H, d, 6.3)	13.4, CH_3_	1.00 (3H, d, 6.7)	11.3, CH_3_	0.93 (3H, d, 7.1)
1′-OH		6.25, s								
3′-OH										7.12 (1H, s)
5′-OH						4.66 (1H, d, 6.2)				4.79 (1H, d, 3.8)
11-OH						4.85 (1H, br, s)				
OCH_3_							53.1, CH_3_	3.29 (3H, s)		
										

*Chaetofanixin B (2).* This is a yellow amorphous powder; [α]25 D –28 (*c* 0.1, CHCl_3_); UV (MeOH) λ_*max*_ 225 (4.69), 252 (3.88), 294 (4.58), 336 (4.28), and 392 (4.81) nm; CD (*c* 0.1, MeOH) λ_*max*_ (Δε) 218 (–4.6), 261 (+0.61), and 326 (–0.98); ^1^H and ^13^C NMR data, see Table 1; HRESIMS m/z 449.1368 [M-H]^–^ (calcd for C_23_H_32_O_5_Cl, 449.1373).

*Chaetofanixin C (3)*. This is a yellow amorphous powder; [α]25 D +30 (*c* 0.1, CHCl_3_); UV (MeOH) λ_*max*_ 219 (4.19), 247 (3.77), 307 (3.48), 386 (4.41), and 455 (2.80) nm; CD (*c* 0.1, MeOH) λ_*max*_ (Δε) 215 (–3.15), 256 (+1.85), 271 (+0.33), 300 (+1.64), 366 (–2.72), and 448 (+0.66) nm; ^1^H and ^13^C NMR data, see Table 1; HRESIMS m/z 449.1362 [M+H]^+^ (calcd for C_23_H_32_O_5_Cl, 449.1362).

*Chaetofanixin D (4)*. This is a yellow amorphous powder; [α]25 D –107 (*c* 0.19, CHCl_3_); UV (MeOH) λ_*max*_ 216 (3.80), 249 (4.22), 307 (3.50), 386 (4.45), and 455 (2.83) nm; CD (*c* 0.1, MeOH) λ_*max*_ (Δε) 229 (–3.50), 261 (+3.16), 370 (–3.31), and 455 (+0.04) nm; ^1^H and^13^C NMR data, see Table 1; HRESIMS m/z 481.1613 [M+H]^+^ (calcd for C_24_H_30_O_8_Cl, 481.1624).

*Chaetofanixin E (5)*. This is a yellow amorphous powder; [α]25 D –25 (*c* 0.2, CHCl_3_); UV (MeOH) λ_*max*_ 210 (5.06), 228 (4.92), 249 (5.05), 270 (4.57), 384 (5.60), and 456 (3.98) nm; CD (*c* 0.1, MeOH) λ_*max*_ (Δε) 200 (+2.97), 219 (–0.24), 236 (+0.24), 271 (–3.63), 303 (–1.92), 337 (–3.59) and 392 (+4.68) nm; ^1^H and ^13^C NMR data, see Table 1; HRESIMS m/z 473.1345 [M+Na]^+^ (calcd for C_23_H_27_O_7_ClNa, 473.1338).

### Pro-angiogenic Activity Experiment

Transgenic zebrafish [Transgenic zebrafish:Tg (flk1:EGFP)] were maintained under conditions of a 14/10-h light/dark cycle at a temperature of 28°C to ensure normal spawning. Healthy male and female mature zebrafish were placed in the breeding tank at a ratio of 1:1. In the next morning, the fertilized eggs were obtained and transferred to zebrafish embryo culture water (containing 5.0 mM NaCl, 0.17 mM KCl, 0.4 mM CaCl_2_, and 0.16 mM MgSO_4_). The zebrafish embryos that have been fertilized for 24 h were treated with 1 mg/mL pronase E solution to remove the egg membrane. Then, they were randomly divided into 12 groups: the normal control group, the model group (vatalanib, PTK787), the positive control group (Danhong injection), and the experimental group (test compounds). Each group has 10 zebrafish embryos, and each group has two parallel repeats. The model group was built based on a significant inhibition of the growth of intersegmental blood vessels (ISVs) by treating zebrafish embryos with vatalanib (0.2 μg/mL PTK787). The test compounds 1–10 (20, 40, and 80 μg/mL) and 10 μl/mL Danhong were added to the 24-well plates with model zebrafish embryos (*n* = 10/well). After incubation in a light-operated incubator at 28 °C for 24 h, the number of ISVs was collected using a fluorescence microscope (SZX16 Tokyo, Japan) (Fan et al., 2015).

### ECD Calculation of Compounds 1–5

Conformational searches were carried out via molecular mechanics with the MM+ method in HyperChem 8.0 software, and the geometries were optimized at the gas-phase B3LYP/6-31G(d) level in Gaussian09 software (Version D.01; Gaussian, Inc.: Wallingford, CT, United States) (Frisch et al., 2013) to afford the energy-minimized conformers. Then, the optimized conformers were subjected to the calculations of ECD spectra using the TD-DFT at BH&HLYP/TZVP, CAM-B3LYP/TZVP, and PEB0/TZVP, and solvent effects of the MeOH solution were evaluated at the same DFT level using the SCRF/PCM method.

## Results and Discussion

**FIGURE 2:**
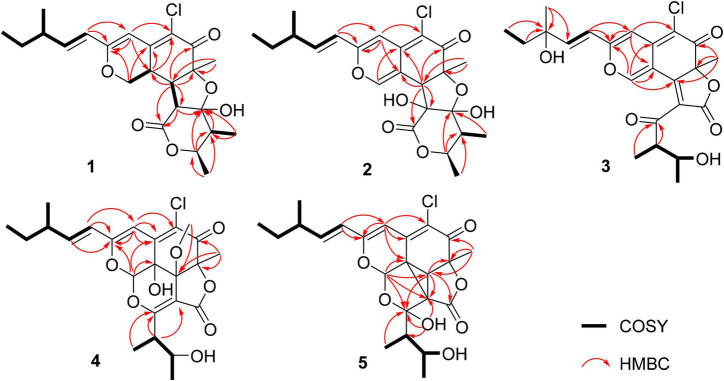
Key ^1^H–^1^ H COSY and HMBC correlations of 1–5.

**FIGURE 3:**
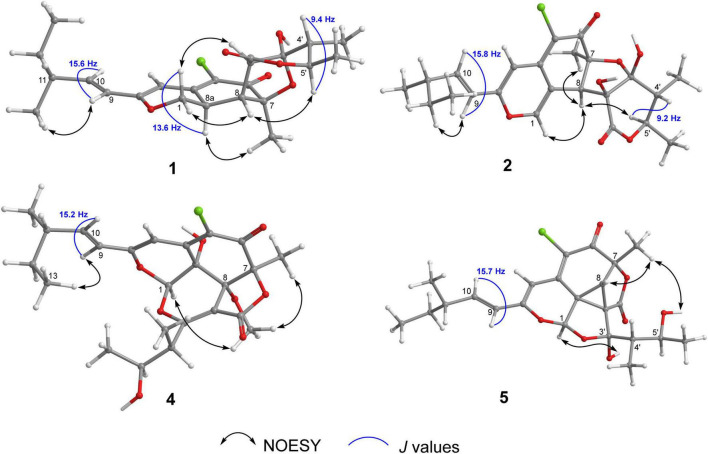
Key NOESY correlations of compounds 1, 2, 4, and 5.

**FIGURE 4:**
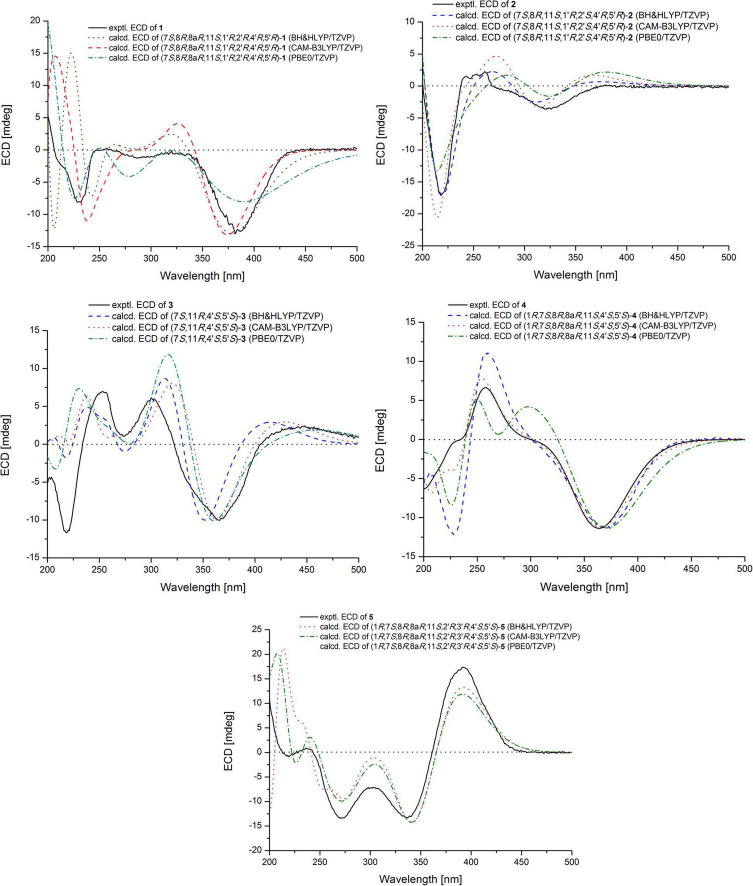
Experimental and calculated ECD spectrum of 1–5.

Chaetofanixin A (1) was obtained as a yellow amorphous powder. The molecular formula of compound 1 was determined to be C_23_H_29_O_6_Cl by HRESIMS (Supplementary Figure 1). The ^1^H and ^13^C NMR data (Table 1), assigned by HSQC, COSY, and HMBC correlations (Figure 2 and Supplementary Figures 4–6), indicated the presence of 23 carbon atoms, which were clarified into five methyls, two methylenes (including one oxygenated), nine methines (including three olefinic and one oxygenated), and seven quaternary carbons (containing two oxygenated, three olefinic, and two carbonyls). Compound 1 has a chlorine atom, which was determined by the HRESIMS spectrum (Supplementary Figure 1) of two isotope peak intensities for [M+H]^+^/[M+H+2]^+^ at a rate of 3:1. Extensive comparison of its 1D and 2D NMR data with those of chaetomugilin D (6) (Qin et al., 2009), a co-metabolite also found in this fungus, revealed that the structures of the two compounds (1 and 6) are very similar, except for the absent double bond (Δ^1,8a^) in 1. Instead, resonances for a methylene group (CH_2_-1, δ_*H/C*_ 4.41, 4.15/68.5) and a methine group (CH-8a, δ_*H/C*_ 3.39/33.3) were observed in the NMR spectra of 1. The above observation indicated that compound 1 was the double-bond hydrogenated derivative of chaetomugilin D (6). Meanwhile, the key COSY correlation (Figure 2 and Supplementary Figure 5) of H_2_-1/H-8a and the HMBC correlations (Figure 2 and Supplementary Figure 6) from H_2_-1 to C-4a and C-8a, H-8a to C-4a and C-5, and H-4 and H-8 to C-8a further confirmed the above deduction. The overall planar structure of 1 was finally defined by the HMBC and COSY correlations (Supplementary Figures 5, 6) as shown in Figure 2. The configuration of the Δ^9^ double bond was assigned as *E* by the coupling constant (15.6 Hz) of H-9/H-10. The large coupling constant (13.6 Hz) of H_β_-1/H-8a indicated their *trans*-diaxial relationship. NOESY correlation (Figure 3 and Supplementary Figure 7) of H_β_-1/H-2′ suggested their same orientation, while correlation of H-8a/CH_3_-7 indicated that they were in the face opposite to H_β_-1. NOESY correlations (Supplementary Figure 7) of H_α_-1/H-8/H-5′ suggested their cofacial relationship. The opposite direction of H-4′ to H-5′ was determined by their large coupling constant (9.4 Hz). From a biosynthetic viewpoint, compound 1 should possess the same absolute configuration at C-11 as 6. The absolute configuration of 1 was assigned as (7*S*,8*R*,8a*R*,11*S*,1′*R*,2′*R*,4′*R*,5′*R*) by its similar ECD curve to that of 6. This was further confirmed by ECD calculation (Figure 4) of (7*S*,8*R*,8a*R*,11*S*,1′*R*,2′*R*,4′*R*,5′*R*)-1, the result of which matched well with the experimental result.

Chaetofanixin B (2) was obtained as a yellow amorphous powder. The molecular formula of compound 2 was established as C_23_H_27_O_7_Cl by HRESIMS (Supplementary Figure 8) peak at m/z 449.1368 [M-H]^–^, as well as ^1^H and ^13^C-DEPTQ NMR data (Table 1 and Supplementary Figures 9, 10), which indicated 10 degrees of unsaturation. A comparison of its ^1^H and ^13^C NMR spectra (Table 1 and Supplementary Figures 9, 10) with those of 6 revealed that their NMR data were very similar. The main difference between them was that signal for a hydroxylated sp^3^ non-protonated carbon of 2 replaced signals for CH-2′ of 6, which indicated that 2 was a hydroxylated derivative of 6 at C-2′. The large *J* value (15.8 Hz) of H-9/H-10 assigned the *E* configuration of the Δ^9^ double bond. The NOESY (Figure 3 and Supplementary Figure 14) correlations of H-8 with CH_3_-7 and H-5′ suggested the cofacial relationship of these protons. The large *J* value (9.2 Hz) of H-4′/H-5′ suggested their *trans*-diaxial relationship. The experimental ECD curve of 2 was similar to that of 6, indicating the (7*S*,8*R*,11*S*,1′*R*,2′*S*,4′*R*,5′*R*) configuration of 2. The ECD calculations (Figure 4) further confirmed this deduction.

Chaetofanixin C (3) was obtained as yellow amorphous powder and was determined to be C_23_H_25_O_7_Cl by HRESIMS (Supplementary Figure 15) peak at m/z 449.1362 [M+H]^+^. The ^1^H and ^13^C-DEPTQ NMR data (Supplementary Figures 16, 17 and Supplementary Table 1), with the aid of HSQC spectra (Supplementary Figure 18), indicated the presence of 23 carbon atoms, which were clarified into five methyls, one methylene, six methines (including four olefinic and one oxygenated), and eleven quaternary carbons (containing two oxygenated, six olefinic, and three carbonyls). An extensive comparison of the above data with those of *epi*-chaetoviridin A (Phonkerd et al., 2008) revealed that the structures of these two compounds are very similar, except that the signals for CH-11 (δ_*H/C*_ 2.29/38.9) in *epi*-chaetoviridin A were absent in the NMR spectra of 3. Instead, a hydroxy group (δ_*H*_ 4.85) and one oxygenated quaternary carbon (δ_*C*_ 72.0) were observed. The above observation indicated that compound 3 was the 11-OH derivative of *epi*-chaetoviridin A. The overall planar structure of 3 was finally defined by the HMBC and COSY correlations (Supplementary Figures 19, 20) as shown in Figure 2. The *J* value (8.6 Hz) of H-4′/H-5′ was similar to those of the two known compounds chaetomugilin Q (7) (Yamada et al., 2011) and chaetoviridin J (8) (Youn et al., 2015), suggesting that the relative configuration of the two chiral centers C-4′ and C-5′ was the same as those of 7 and 8. The absolute configurations of C-7, C-4′, and C-5′ of 3 were proposed to be the same as its cometabolites 1, 2, and 6–8 from biosynthetic considerations, and were further confirmed by ECD calculations (Figure 4). However, the absolute configuration of C-11 remains unassigned.

Chaetofanixin D (4) was obtained as a yellow amorphous powder. The molecular formula of compound 4 was established as C_24_H_29_O_8_Cl by HRESIMS (Supplementary Figure 21) peak at m/z 481.1613 [M+H]^+^, as well as ^1^H and ^13^C-DEPTQ NMR data (Table 1 and Supplementary Figures 22, 23), which indicated 10 degrees of unsaturation. The ^13^C-DEPTQ and HSQC NMR spectra (Supplementary Figures 23, 24) revealed the presence of 24 carbons, which were clarified into six methyls (including one oxygenated), one methylene, seven methines (including three olefinic and two oxygenated), and ten quaternary carbons (containing five olefinic, three oxygenated, and two carbonyls). Comparison of its ^1^H and ^13^C NMR spectra (Table 1 and Supplementary Figures 22, 23) with those of the known azaphilone, chaetoviridin I (9) (Borges et al., 2011), which was also isolated from the culture broth of YP-6, revealed that their NMR data were very similar, except that additional signals for an oxygenated methyl (CH_3_O-8, δ_*H/C*_ 3.29/53.1) were observed in the NMR spectra of 4. The key HMBC correlation (Figure 2 and Supplementary Figure 26) of CH_3_O-8 to C-8 revealed the location of this oxygenated methyl at C-8 in 4. The similarity of the coupling constants and chemical shifts between 4 and 9,^17^ as well as NOESY (Figure 3) correlations from CH_3_O-8 to CH_3_-7 and H-1, indicated their same relative configuration. The optical rotation value (–107) and experimental ECD curve were very similar to those of 9 (–37), suggesting their same (1*R*,7*S*,8*R*,8a*R*,11*S*,4′*S*,5′*S*) absolute configuration. The calculated ECD spectrum for the (1*R*,7*S*,8*R*,8a*R*,11*S*,4′*S*,5′*S*)-4 matched well with the experimental curve, further confirming the above deduction (Figure 4).

Chaetofanixin E (5) was obtained as a yellow amorphous powder. Its molecular formula was determined as C_23_H_27_O_7_Cl based on the HRESIMS peak at m/z 473.1345 [M+Na]^+^ (Supplementary Figure 28) and ^1^H and ^13^C-DEPTQ NMR data (Table 1 and Supplementary Figures 29, 30), which indicated 10 degrees of unsaturation. Two isotope peak intensities for [M+H]^+^/[M+H+2]^+^ at a rate of 3:1 indicated the presence of chlorine atom in compound 5. The ^13^C NMR spectrum showed 23 signals that were classified by HSQC (Supplementary Figure 31) as nine non-protonated carbons (two keto carbonyls, three olefinic carbons, and two oxygenated ones), two sp^2^-methine carbons, six sp^3^-methine carbons (two oxygenated ones), one methylene carbon, and five methyl carbons. The ^1^H-NMR spectrum showed five methyl signals at δ_*H*_ 0.85 (d, *J* = 7.4 Hz), 0.93 (d, *J* = 7.1 Hz), 1.55 (s), 1.02 (d, *J* = 6.8 Hz), and 1.03 (d, *J* = 6.6 Hz); three olefin signals at δ_*H*_ 6.09 (s), 6.19 (d, *J* = 15.7 Hz), and 6.39 (dd, *J* = 15.6, 7.9 Hz); and two oxygenated signals at δ_*H*_ 5.74 (s) and 3.70 (m). Further analysis of its NMR data suggested that chaetofanixin E (5) is similar to chaetoviridin I (9). The comparison of NMR data with those of 9 revealed that two oxygenated non-protonated carbons (C-8/C-8a) and a double bond (Δ^2’,3’^) of chaetoviridin I (9) were replaced by an sp^3^-methine carbon (CH-8, δ_*H/C*_ 2.93/39.1), two non-protonated sp^3^ carbons (C-8a, δ_*C*_ 39.2; C-2′, δ_*C*_ 55.0), and an oxygenated non-protonated carbon (C-3′, δ_*C*_ 107.8) of compound 5. In the HMBC spectrum (Figure 2 and Supplementary Figure 33), both H-1 and H-8 correlated with two quaternary carbons C-8a at δ_*C*_ 39.2 and C-2′ at δ_*C*_ 55.0, indicating the presence of a direct linkage between C-8a and C-2′. HMBC correlations (Figure 2 and Supplementary Figure 33) from CH_3_-4′ to C-4′, C-5′, and the deoxygenated non-protonated carbon C-3′ indicated the presence of a hydroxy group at C-3. Thus, compound 5 was assigned to be a novel member of the chloro*-*azaphilone family, the structure of which bears a highly rigid 6/6/5/3/5 penta-cyclic ring system. The large coupling constant (*J*_9/10_ = 15.7 Hz) in the ^1^H-NMR data (Table 1 and Supplementary Figure 29) deduced the configuration of a double bond at C-9/C-10 as *trans* form. The NOESY (Figure 3 and Supplementary Figure 34) correlations from H-8/CH_3_-7/OH-5′ and H-1/OH-3′, with the aid of biosynthetic analysis from other cometabolites, led to the assignment of the relative configuration of 5 as shown in Figure 1. The calculated ECD spectrum (Figure 4) for the (1*R*,7*S*,8*R*,8a*R*,11*S*,2′*R*,3′*R*,4′*S*,5′*S*)-5 matched well with that of the experimental curve, allowing the establishment of the absolute configuration of 5 (Figure 1). Therefore, the chiral centers of compound 5 were tentatively assigned as (1*R*,7*S*,8*R*,8a*R*,11*S*,2′*R*,3′*R*,4′*S*,5′*S*).

**FIGURE 5:**
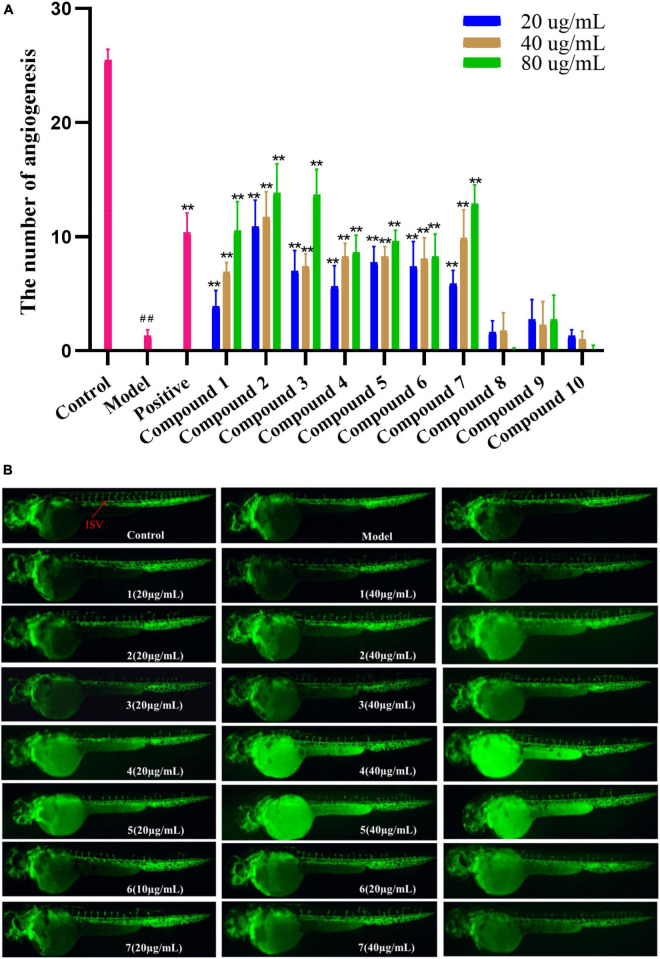
**(A)** Vasculogenesis activity of compounds 1–10 (20, 40, and 80 μg/mL). ^##^*P* < 0.01, compared with the normal control group; **P < 0.01, compared with the model control group. **(B)** Images of intersomitic vessels (ISV) in transgenic fluorescent zebrafish.

Compounds 1–10 were evaluated for pro-angiogenic activity in the zebrafish models at concentrations of 20, 40, and 80 μg/mL. Compared with the model group, 1–7 significantly promoted angiogenesis in a dose-dependent manner. Among them, compounds 2, 3, and 7 showed potent pro-angiogenic activities at the concentration of 80 μg/mL, while compounds 8-10 did not show relevant activities (Figure 5). In addition, the activity of compound 2 was stronger than those of 6 and 10, suggesting that hydroxylation at C-2′ and C-3′ might contribute to the pro-angiogenic activity. Compound 4 exhibited much higher activity than that of 9, implying that methylation at 8-OH might increase the activity. Furthermore, compound 7 showed stronger activities than 8, indicating that oxidation at C-12 could increase the activity. The results suggested that compounds 1–7 could be promising candidates for the development of lead drugs against cardiovascular diseases.

In summary, five new chloro-azaphilone derivatives, including a structurally unique one, and five related known analogs were isolated from the culture extract of *Chaetomium globosum* YP-106, a fungus obtained from the hadal trench-derived seawater. Compounds 1–7 showed excellent pro-angiogenic activities in a dose-dependent manner, possessing the potential to be developed as natural cardiovascular disease agents. These results further demonstrated the great potential for the development and utilization of deep-sea microbial resources for drug research.

## Data Availability Statement

The original contributions presented in this study are included in the article/Supplementary Material, further inquiries can be directed to the corresponding authors.

## Ethics Statement

The animal study was reviewed and approved by Ethics Committee of the Biology Institute of Shandong Academy of Science. Written informed consent was obtained from the owners for the participation of their animals in this study.

## Author Contributions

YF conceived and designed the experiments and wrote the manuscript. CJ, YZ, and ZM contributed to the isolation of compounds. PL and TF contributed to the bioactivity assay. LG revised the manuscript. LZ and LX supervised the work. All authors contributed to the article and approved the submitted version.

## Conflict of Interest

The authors declare that the research was conducted in the absence of any commercial or financial relationships that could be construed as a potential conflict of interest.

## Publisher’s Note

All claims expressed in this article are solely those of the authors and do not necessarily represent those of their affiliated organizations, or those of the publisher, the editors and the reviewers. Any product that may be evaluated in this article, or claim that may be made by its manufacturer, is not guaranteed or endorsed by the publisher.

## References

[B1] Borges W. S. Mancilla G. Guimarães D. O. Durán-Patrón R. Collado I. G. Pupo M. T. (2011). Azaphilones from the endophyte *Chaetomium globosum*. *J. Nat. Prod.* 74 1182–1187. 10.1021/np200110f 21548578

[B2] Carroll A. R. Copp B. R. Davis R. A. Keyzers R. A. Prinsep M. R. (2021). Marine natural products. *Nat. Prod. Rep.* 38 362–413. 10.1039/D0NP00089B 33570537

[B3] Chi L. P. Li X. M. Wan Y. P. Li X. Wang B. G. (2020b). Ophiobolin sesterterpenoids and farnesylated phthalide derivatives from the deep sea cold-seep-derived fungus *Aspergillus insuetus* SD-512. *J. Nat. Prod.* 83 3652–3660. 10.1021/acs.jnatprod.0c00860 33322904

[B4] Chi L. P. Li X. M. Li L. Li X. Wang B. G. (2020a). Cytotoxic thiodiketopiperazine derivatives from the deep sea-derived fungus *Epicoccum nigrum* SD-388. *Mar. Drugs* 18:160. 10.3390/md18030160 32183021 PMC7143119

[B5] Fan Y. Q. Li P. H. Chao Y. X. Chen H. Du N. He Q. X. (2015). Alkaloids with cardiovascular effects from the marine-derived fungus *Penicillium expansum* Y32. *Mar. Drugs* 13 6489–6504. 10.3390/md13106489 26506361 PMC4626702

[B6] Frisch M. J. Trucks G. W. Schlegel H. B. Scuseria G. E. Robb M. A. Cheeseman J. R. (2013). *Gaussian 09, Version D. 01.*

[B7] Guo X. Meng Q. Niu S. Liu J. Guo X. Sun Z. (2021). Epigenetic manipulation to trigger production of guaiane-type sesquiterpenes from a marine-derived *Spiromastix* sp. fungus with antineuroinflammatory effects. *J. Nat. Prod.* 84 1993–2003. 10.1021/acs.jnatprod.1c00293 34161733

[B8] Jude E. B. Eleftheriadou I. Tentolouris N. (2010). Peripheral arterial disease in diabetes-a review. *Diabetic Med.* 27 4–14. 10.1111/j.1464-5491.2009.02866.x 20121883

[B9] Kurusamy S. López-Maderuelo D. Little R. Cadagan D. Savage A. M. Ihugba J. C. (2017). Selective inhibition of plasma membrane calcium ATPase 4 improves angiogenesis and vascular reperfusion. *J. Mol. Cell. Cardiol.* 109 38–47. 10.1016/j.yjmcc.2017.07.001 28684310

[B10] Li P. Zhang M. Xie D. Zhang X. Zhang S. Gao F. (2021). Characterization and bioactivities of phospholipids from squid viscera and gonads using ultra-performance liquid chromatography-Q-exactive orbitrap/mass spectrometry-based lipidomics and zebrafish models. *Food Funct.* 12 7986–7996. 10.1039/D1FO00796C 34259702

[B11] Phonkerd N. Kanokmedhakul S. Kanokmedhakul K. Soytong K. Prabpai S. Kongsearee P. (2008). Bis-spiro-azaphilones and azaphilones from the fungi *Chaetomium cochliodes* VTh01 and C. cochliodes CTh05. *Tetrahedron* 64 9636–9645. 10.1016/j.tet.2008.07.040

[B12] Potz B. A. Parulkar A. B. Abid R. M. Sodha N. R. Sellke F. W. (2017). Novel molecular targets for coronary angiogenesis and ischemic heart disease. *Coronary Artery Dis.* 28 605–613. 10.1097/MCA.0000000000000516 28678145 PMC5624824

[B13] Qin J. C. Zhang Y. M. Gao J. M. Bai M. S. Yang S. X. Laatsch H. (2009). Bioactive metabolites produced by *Chaetomium globosum*, an endophytic fungus isolated from *Ginkgo biloba*. *Bioorg. Med. Chem. Lett.* 19 1572–1574. 10.1016/j.bmcl.2009.02.025 19246197

[B14] Skropeta D. Wei L. (2014). Recent advances in deep-sea natural products. *Nat. Prod. Rep.* 31 999–1025. 10.1039/B808743A 24871201

[B15] Virani S. S. Alonso A. Benjamin E. J. Bittencourt M. S. Callaway C. W. Carson A. P. (2020). Heart disease and stroke statistics—2020 update: a report from the American heart association. *Circulation* 141 e139–e596. 10.1161/CIR.0000000000000757 31992061

[B16] Wang J. He W. Huang X. Tian X. Liao S. Yang B. (2016). Antifungal new oxepine-containing alkaloids and xanthones from the deep-sea-derived fungus *Aspergillus versicolor* SCSIO 05879. *J. Agric. Food Chem.* 14 2910–2916. 10.1021/acs.jafc.6b00527 26998701

[B17] Wang S. Li X. M. Teuscher F. Li D. L. Diesel A. Ebel R. (2006). Chaetopyranin, a benzaldehyde derivative, and other related metabolites from *Chaetomium globosum*, an endophytic fungus derived from the marine red alga *Polysiphonia urceolata*. *J. Nat. Prod.* 69 1622–1625. 10.1021/np060248n 17125234

[B18] Wang Y. N. Meng L. H. Wang B. G. (2020). Progress in research on bioactive secondary metabolites from deep-sea derived microorganisms. *Mar. Drugs* 18:614. 10.3390/md18120614 33276592 PMC7761599

[B19] Yamada T. Muroga Y. Jinno M. Kajimoto T. Usami Y. Numata A. (2011). New class azaphilone produced by a marine fish-derived *Chaetomium globosum*. the stereochemistry and biological activities. *Bioorg. Med. Chem.* 19 4106–4113. 10.1016/j.bmc.2011.05.008 21640594

[B20] Yan L. H. Li P. H. Li X. M. Yang S. Q. Liu K. C. Wang B. G. (2022). Chevalinulins A and B, proangiogenic alkaloids with a spiro[bicyclo[2.2.2]octane-diketopiperazine] skeleton from deep sea cold-seep-derived fungus *Aspergillus chevalieri* CS-122. *Org. Lett.* 24 2684–2688. 10.1021/acs.orglett.2c00781 35389665

[B21] Youn U. J. Sripisut T. Park E. J. Kondratyuk T. P. Fatima N. Simmons C. J. (2015). Determination of the absolute configuration of chaetoviridins and other bioactive azaphilones from the endophytic fungus *Chaetomium globosum*. *Bioorg. Med. Chem. Lett.* 25 4719–4723. 10.1016/j.bmcl.2015.08.063 26343828

[B22] Zhou F. Dai O. Peng C. Xiong L. Ao H. Liu F. (2021). Pro-angiogenic effects of essential oil from perilla frutescens and its main component (perillaldehyde) on zebrafish embryos and human umbilical vein endothelial cells. *Drug Des. Dev. Ther.* 15 4985–4999. 10.2147/DDDT.S336826 34924753 PMC8674578

