# Supplementary material for: Pro-angiogenic New Chloro-Azaphilone Derivatives From the Hadal Trench-Derived Fungus Chaetomium globosum YP-106
Source: Front Microbiol. 2022 Jul 22;13:943452. doi: 10.3389/fmicb.2022.943452 (PMC9355395; doi:10.3389/fmicb.2022.943452)
Supplement: Supplementary file 1 [file Data_Sheet_1.PDF]

Supporting Information

Pro-angiogenic new chloro-azaphilone derivatives from the hadal trench-derived fungus *Chaetomium globosum* YP-106.

Yaqin Fan[#,1], Chunjiao Jiang[#,1], Zhiheng Ma[1], Yan Zhang[1], Peihai Li[2], Lizhong Guo[1], Ting Feng[3], Liman Zhou[3,*], Lili Xu[1,*]

[1]*Shandong Provincial Key Laboratory of Applied Mycology, School of Life Sciences, Qingdao Agricultural University, Qingdao 266109, People's Republic of China.*

[2]*Engineering Research Center of Zebrafish Models for Human Diseases and Drug Screening of Shandong Province, Shandong Provincial Engineering Laboratory for Biological Testing Technology, Key Laboratory for Biosensor of Shandong Province, Biology Institute, Qilu University of Technology (Shandong Academy of Sciences), Jinan 250103, People's Republic of China.*

[3]*Key Laboratory of Chemistry and Engineering of Forest Products, State Ethnic Affairs Commission, Guang-xi Key Laboratory of Chemistry and Engineering of Forest Products, Guangxi Collaborative Innovation Center for Chemistry and Engineering of Forest Products, School of Chemistry and Chemical Engineering, Guangxi Minzu University, Nanning 530006, People's Republic of China.*

[#] *Yaqin Fan and Chunjiao Jiang contributed equally to this work.*

Table of Contents

Contents	page
**Figure S1**. HRESIMS spectrum of chaetofanixin A (**1**)	S4
**Figure S2**. ^{1}H-NMR spectrum of chaetofanixin A (**1**) in DMSO-d_6	S5
**Figure S3**. ^{13}C- NMR spectrum of chaetofanixin A (**1**) in DMSO-d_6	S6
**Figure S4**. HSQC spectrum of chaetofanixin A (**1**) in DMSO-d_6	S7
**Figure S5**. ^{1}H-^{1}H COSY spectrum of chaetofanixin A (**1**) in DMSO-d_6	S8
**Figure S6**. HMBC spectrum of chaetofanixin A (**1**) in DMSO-d_6	S9
**Figure S7**. NOESY spectrum of chaetofanixin A (**1**) in DMSO-d_6	S10
**Figure S8**. HRESIMS spectrum of chaetofanixin B (**2**)	S11
**Figure S9**. ^{1}H-NMR spectrum of chaetofanixin B (**2**) in DMSO-d_6	S12
**Figure S10**. ^{13}C-DEPTQ spectrum of chaetofanixin B (**2**) in DMSO-d_6	S13
**Figure S11**. HSQC spectrum of chaetofanixin B (**2**) in DMSO-d_6	S14
**Figure S12**. ^{1}H-^{1}H COSY spectrum of chaetofanixin B (**2**) in DMSO-d_6	S15
**Figure S13**. HMBC spectrum of chaetofanixin B (**2**) in DMSO-d_6	S16
**Figure S14**. NOESY spectrum of chaetofanixin C (**2**) in DMSO-d_6	S17
**Figure S15**. HRESIMS spectrum of chaetofanixin C (**3**)	S19
**Figure S16**. ^{1}H-NMR spectrum of chaetofanixin C (**3**) in DMSO-d_6	S20
**Figure S17**. ^{13}C-DEPTQ spectrum of chaetofanixin C (**3**) in DMSO-d_6	S21
**Figure S18**. HSQC spectrum of chaetofanixin C (**3**) in DMSO-d_6	S22
**Figure S19**. ^{1}H-^{1}H COSY spectrum of chaetofanixin C (**3**) in DMSO-d_6	S23
**Figure S20**. HMBC spectrum of chaetofanixin C (**3**) in DMSO-d_6	S24
**Figure S21**. HRESIMS spectrum of chaetofanixin D (**4**)	S25
**Figure S22**. ^{1}H-NMR spectrum of chaetofanixin D (**4**) in DMSO-d_6	S26
**Figure S23**. ^{13}C-DEPTQ spectrum of chaetofanixin D (**4**) in DMSO-d_6	S27
**Figure S24**. HSQC spectrum of chaetofanixin D (**4**) in DMSO-d_6	S28
**Figure S25**. ^{1}H-^{1}H COSY spectrum of chaetofanixin D (**4**) in DMSO-d_6	S29
**Figure S26**. HMBC spectrum of chaetofanixin D (**4**) in DMSO-d_6	S30
**Figure S27**. NOESY spectrum of chaetofanixin D (**4**) in DMSO-d_6	S31
**Figure S28**. HRESIMS spectrum of chaetofanixin E (**5**)	S32

Figure S29. ^{1}H-NMR spectrum of chaetofanixin E (**5**) in DMSO-d_6 S33
Figure S30. ^{13}C-DEPTQ spectrum of chaetofanixin E (**5**) in DMSO-d_6 S34
Figure S31. HSQC spectrum of chaetofanixin E (**5**) in DMSO-d_6 S35
Figure S32. ^{1}H-^{1}H COSY spectrum of chaetofanixin E (**5**) in DMSO-d_6 S36
Figure S33. HMBC spectrum of chaetofanixin E (**5**) in DMSO-d_6 S37
Figure S34. NOESY spectrum of chaetofanixin E (**5**) in DMSO-d_6 S38

Figure S1. HRESIMS spectrum of chaetofanixin A (**1**)

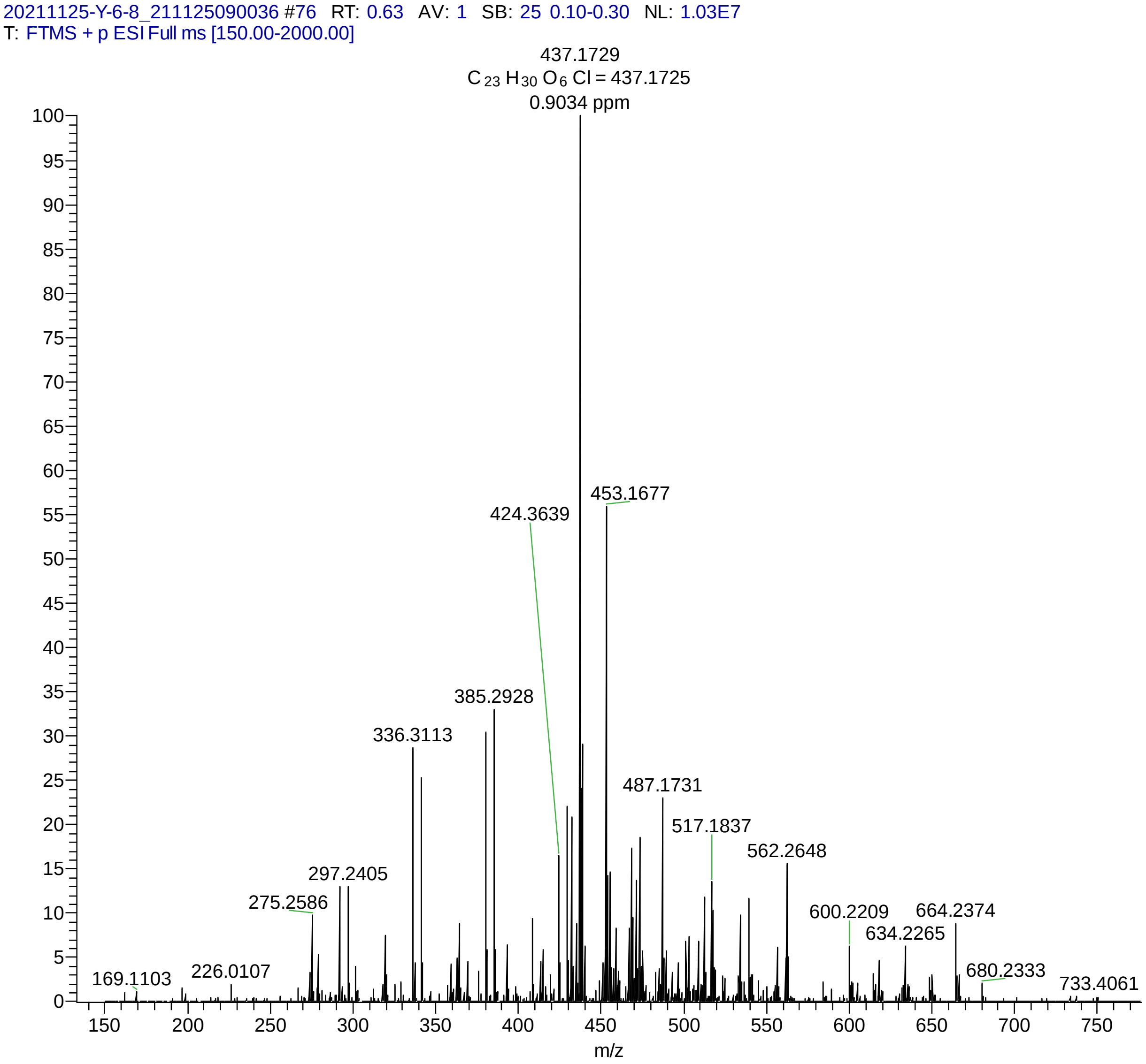

Figure S2. ^{1}H-NMR spectrum of chaetofanixin A (**1**) in DMSO-d_6

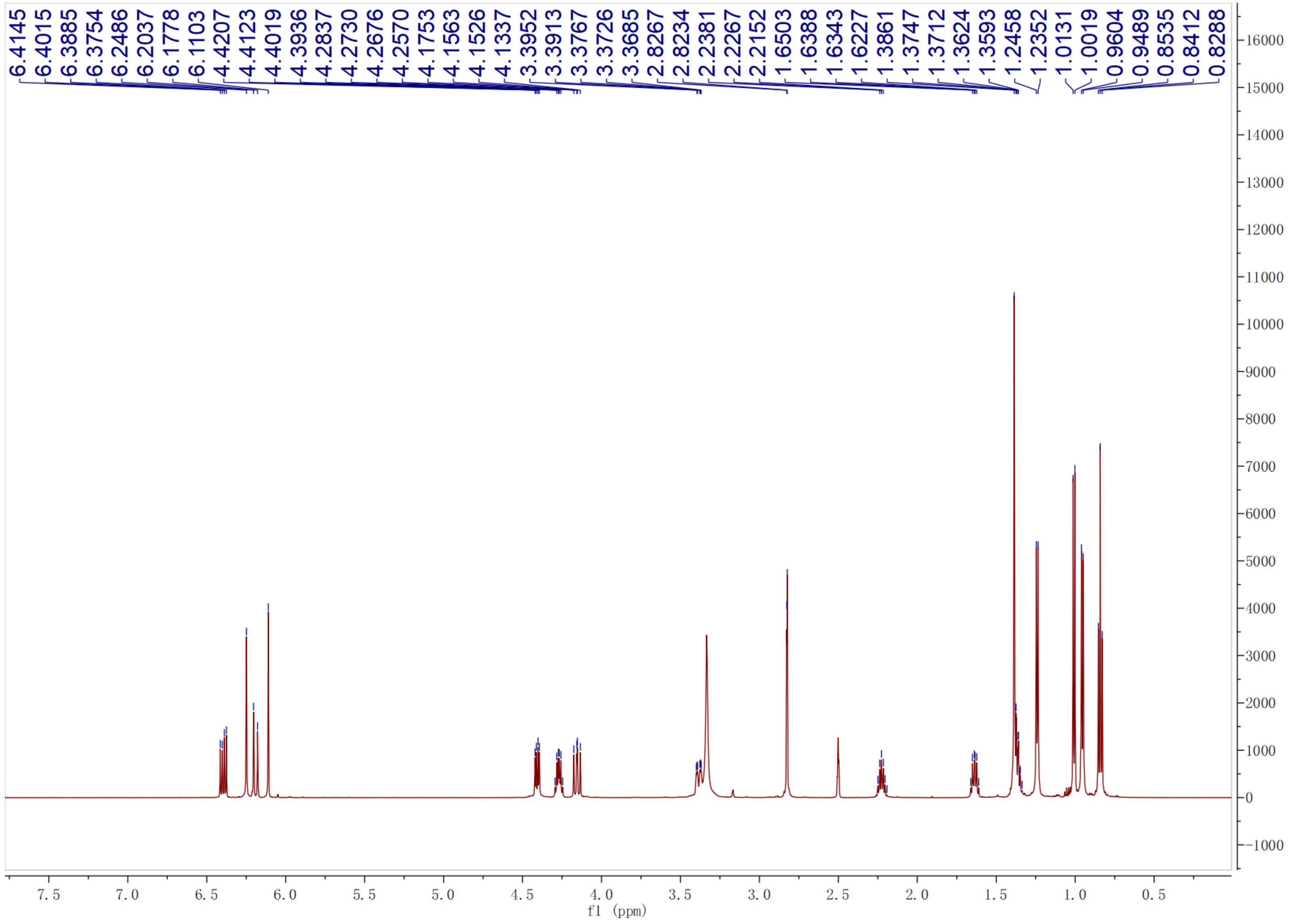

Figure S3. ^{13}C- NMR spectrum of chaetofanixin A (**1**) in DMSO-d_6

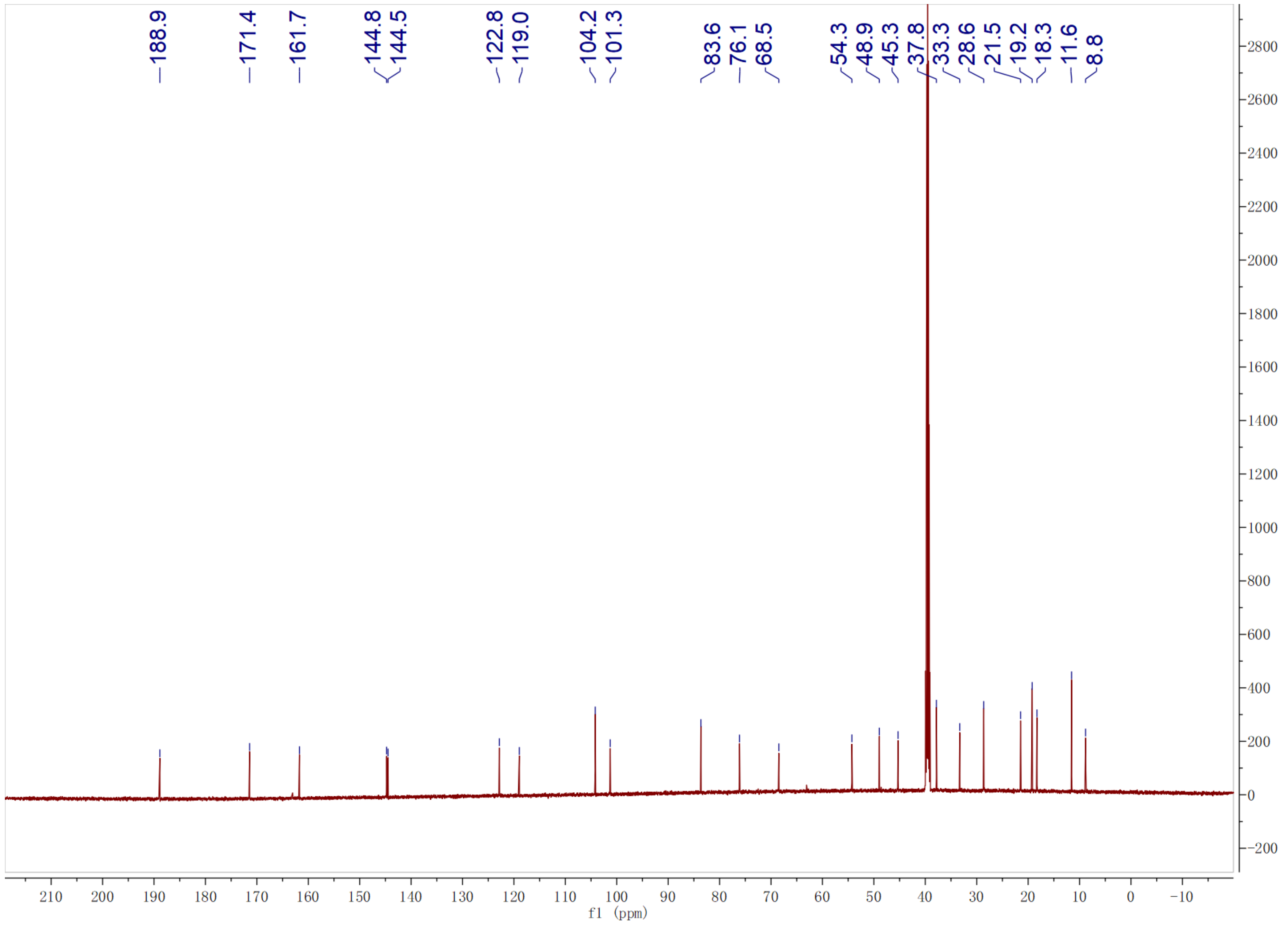

Figure S4. HSQC spectrum of chaetofanixin A (**1**) in DMSO-d_6

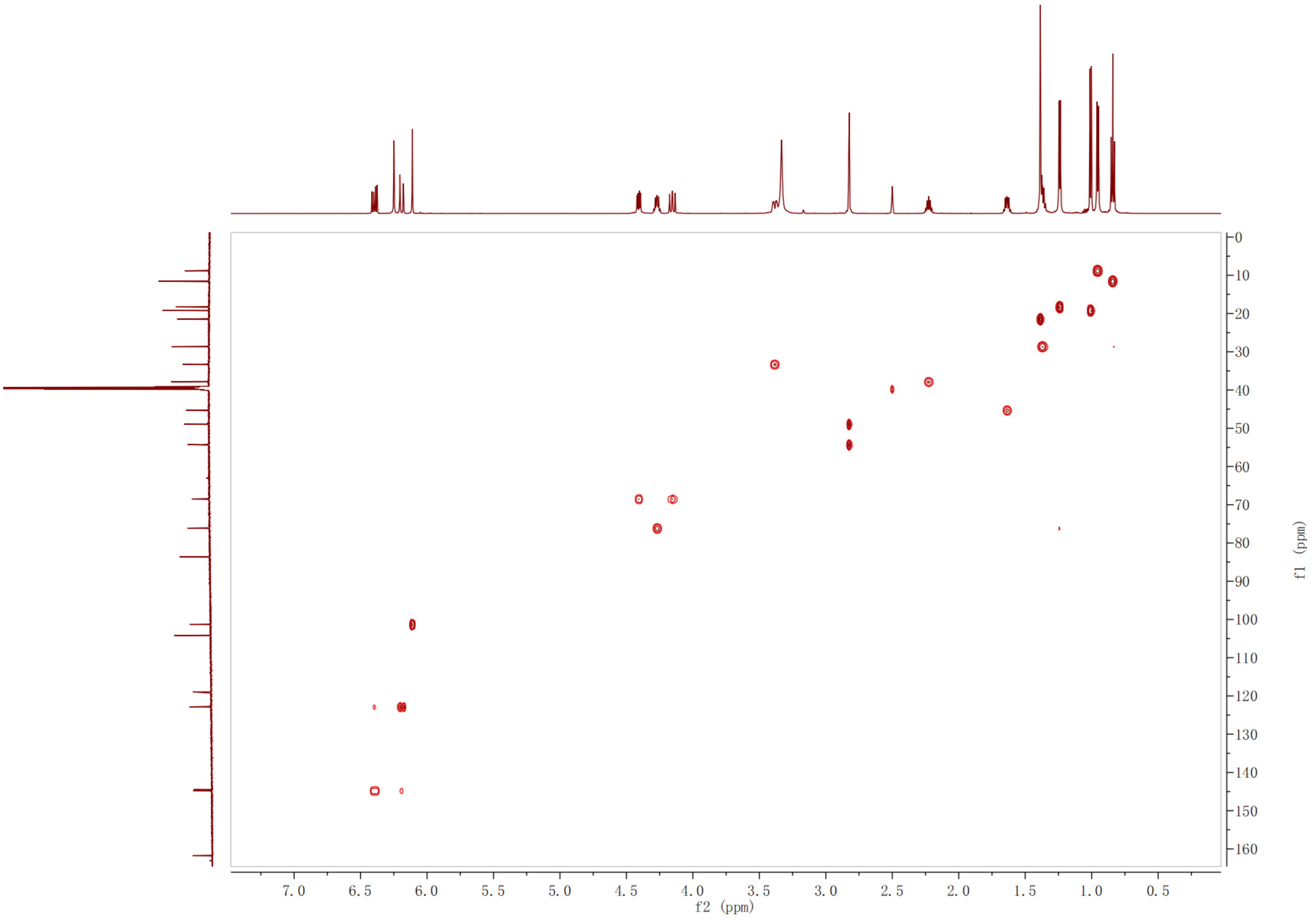

Figure S5. ^{1}H-^{1}H COSY spectrum of chaetofanixin A (**1**) in DMSO-d_6

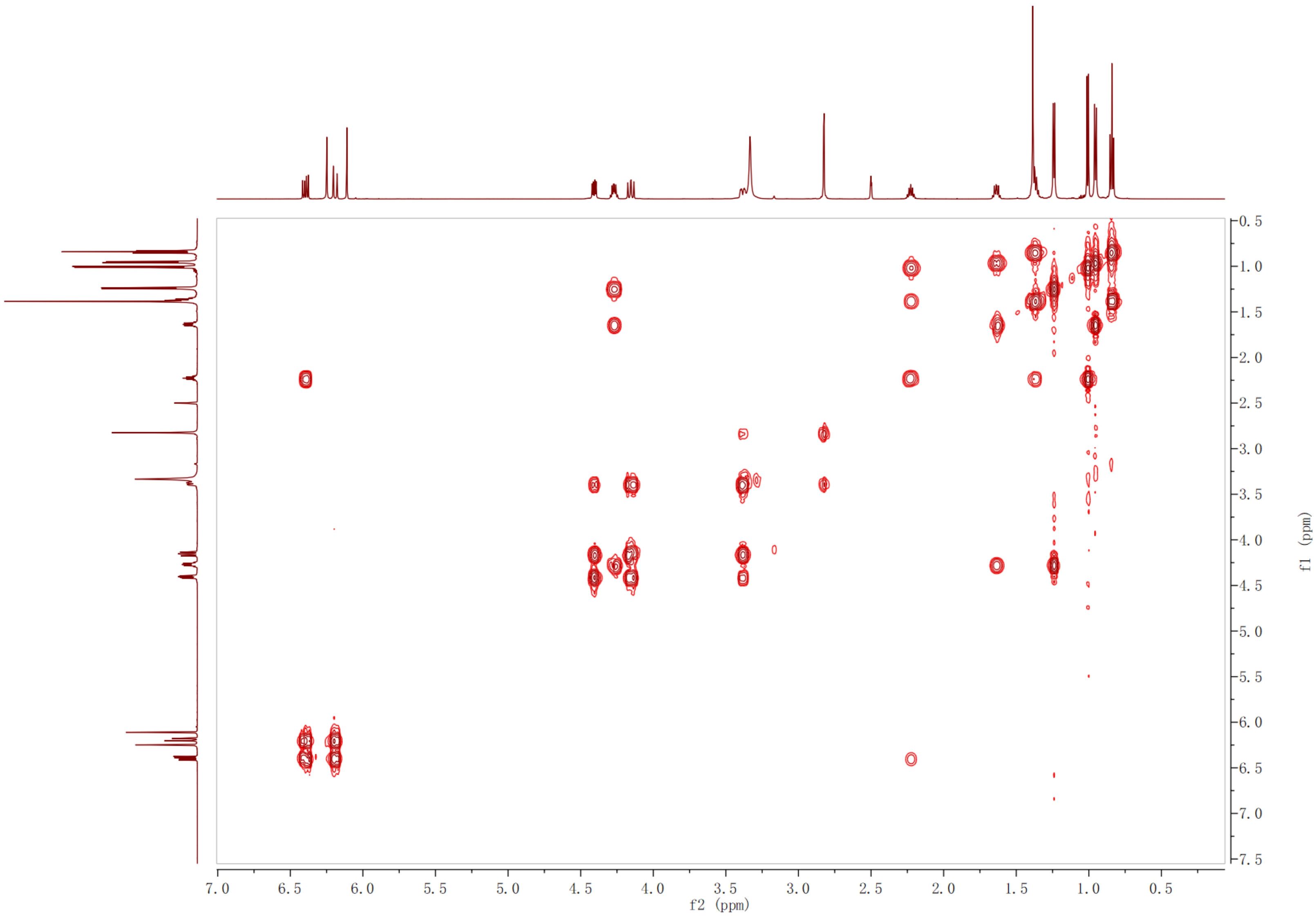

Figure S6. HMBC spectrum of chaetofanixin A (**1**) in DMSO-d_6

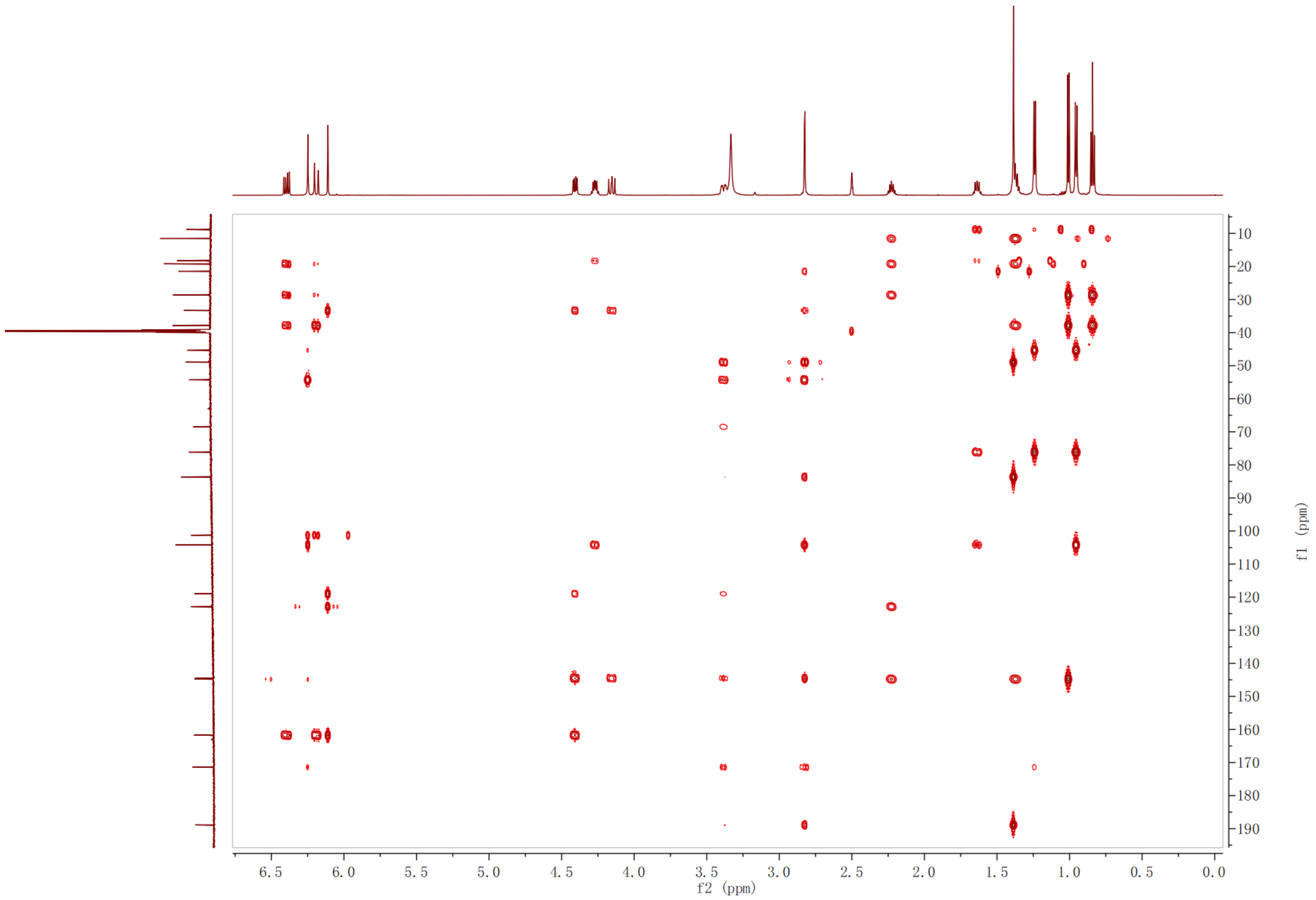

Figure S7. NOEZY spectrum of chaetofanixin A (**1**) in DMSO-d_6

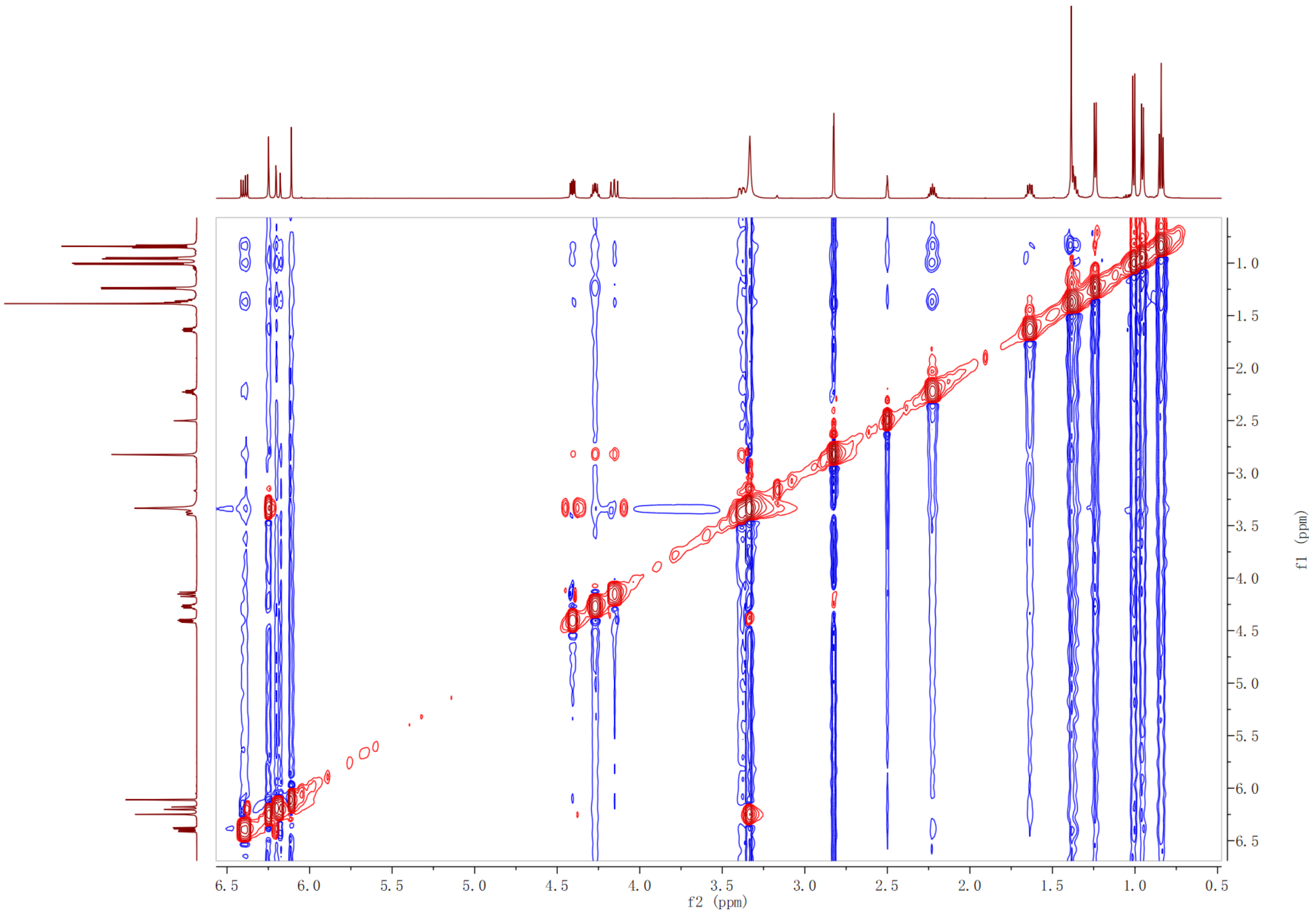

Figure S8. HRESIMS spectrum of chaetofanixin B (**2**)

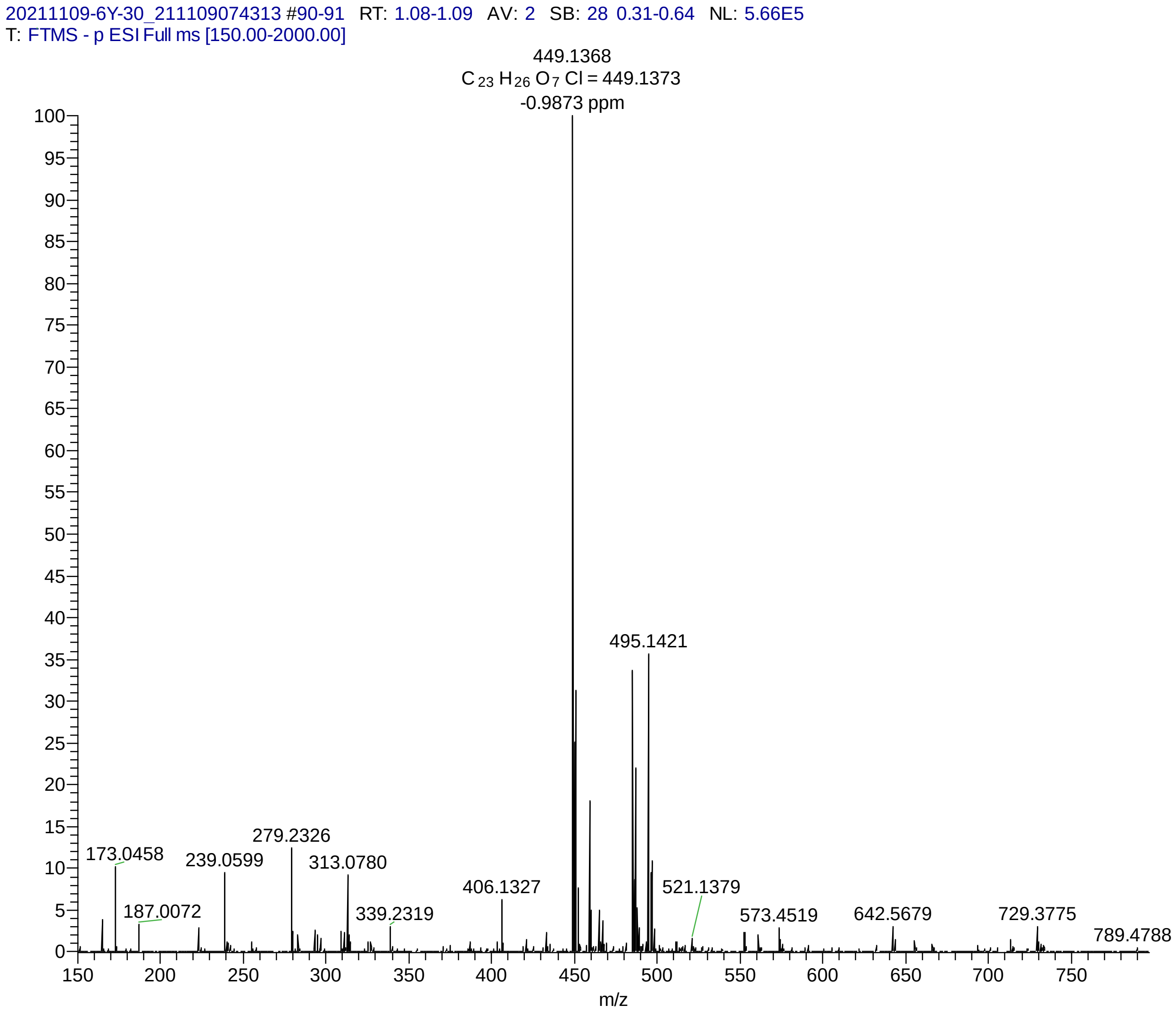

Figure S9. ^{1}H-NMR spectrum of chaetofanixin B (**2**) in DMSO-d_6

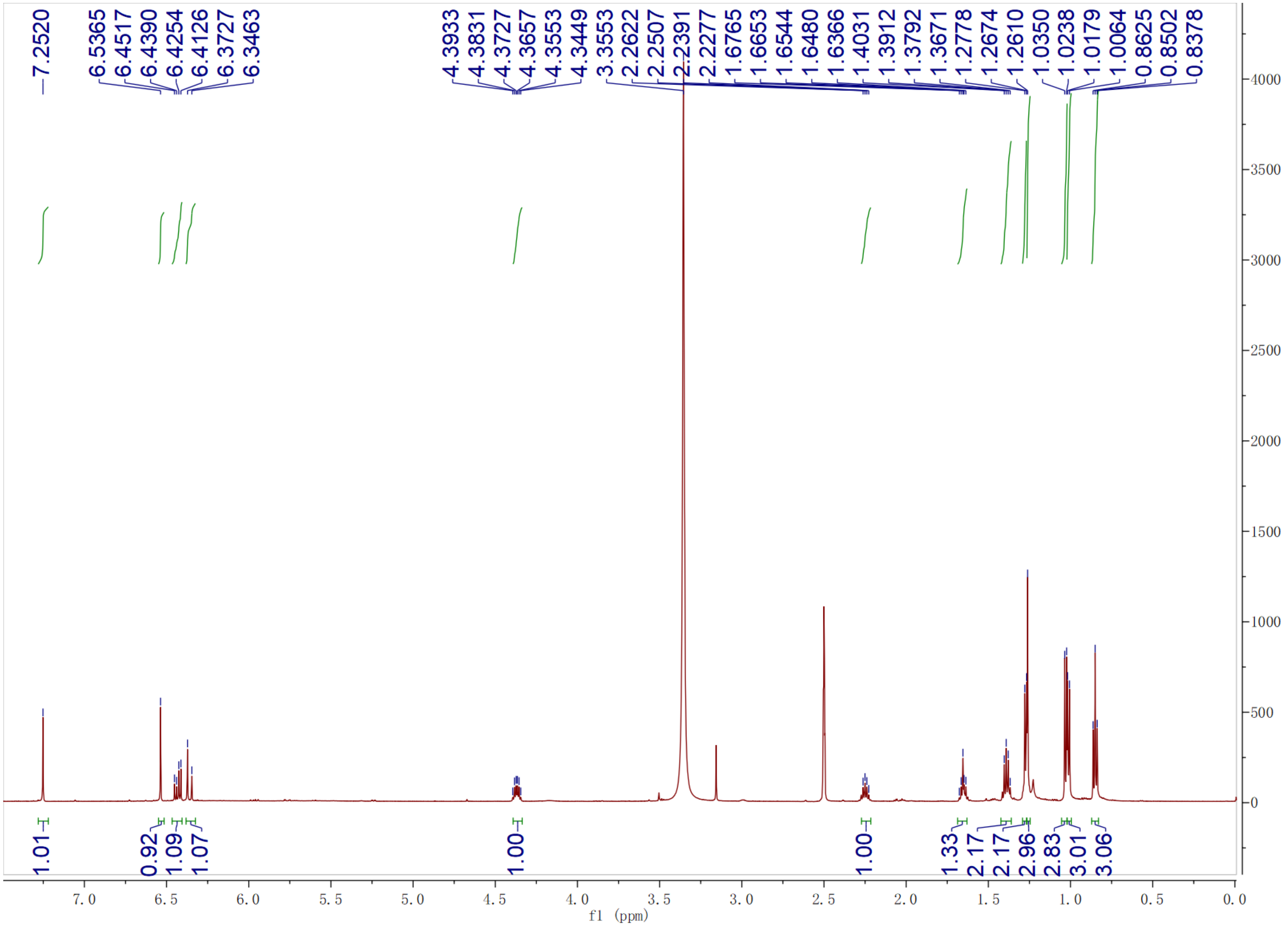

Figure S10. ^{13}C-DEPTQ spectrum of chaetofanixin B (**2**) in DMSO-d_6

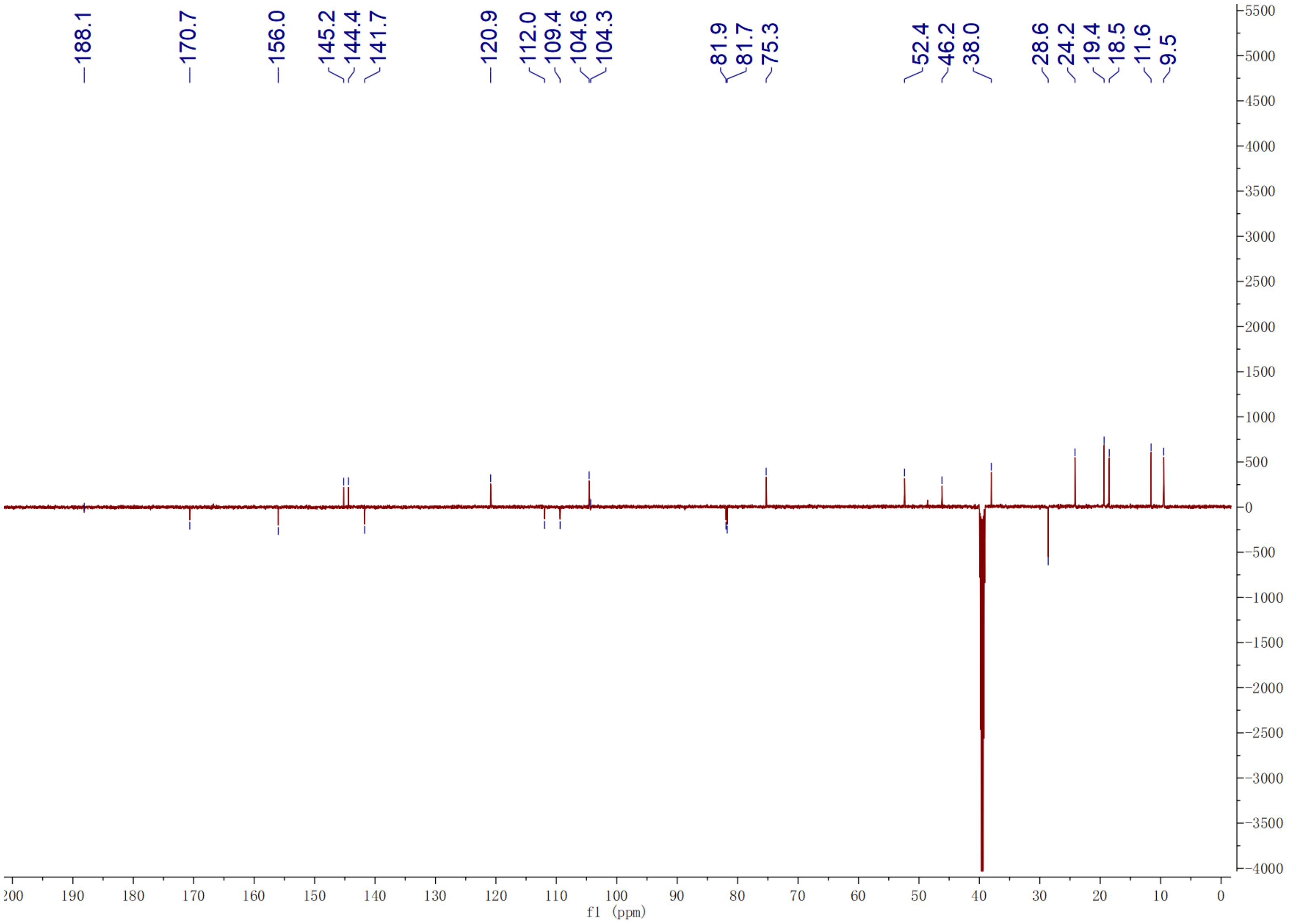

Figure S11. HSQC spectrum of chaetofanixin B (**2**) in DMSO-d_6

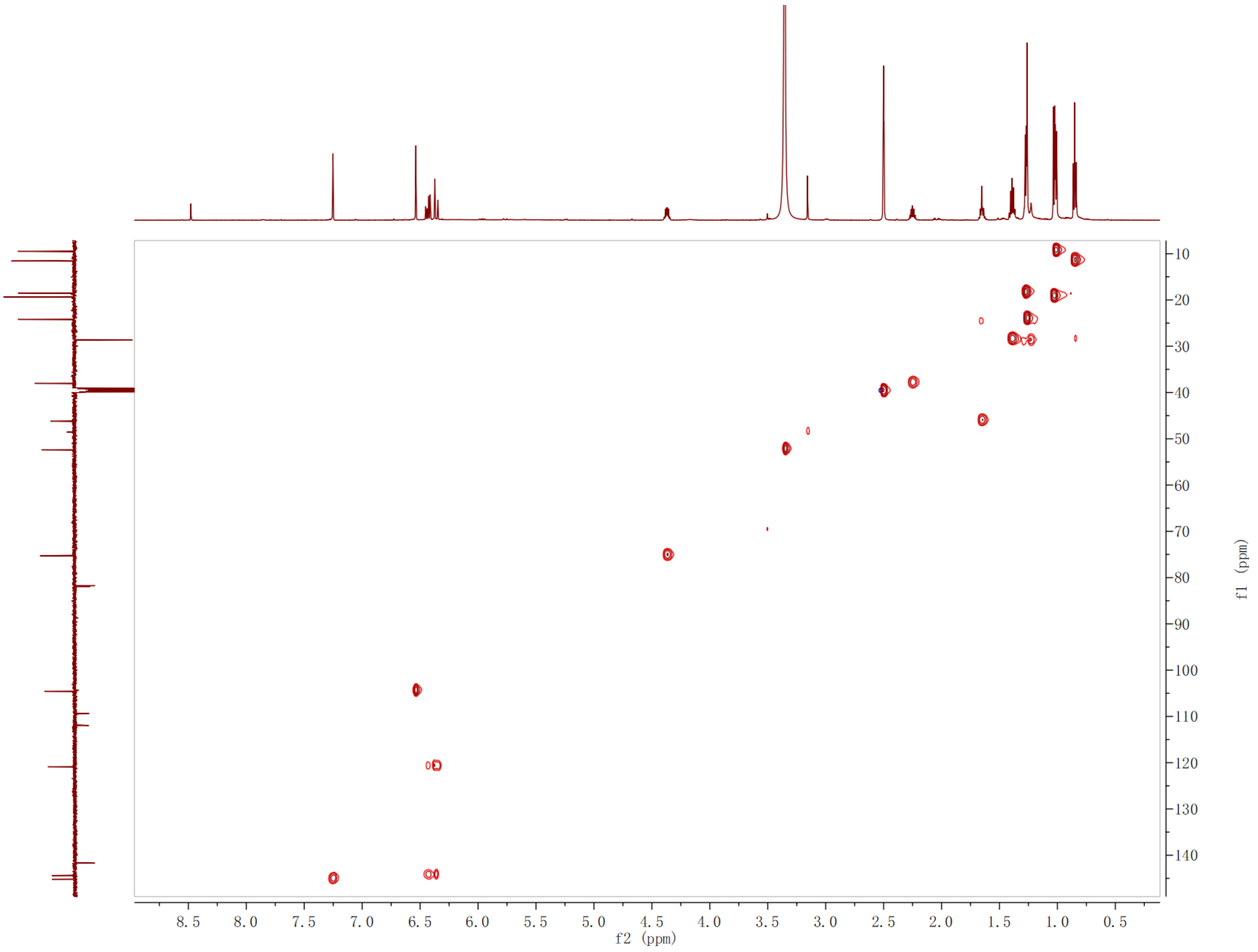

Figure S12. ^{1}H-^{1}H COSY spectrum of chaetofanixin B (**2**) in DMSO-d_6

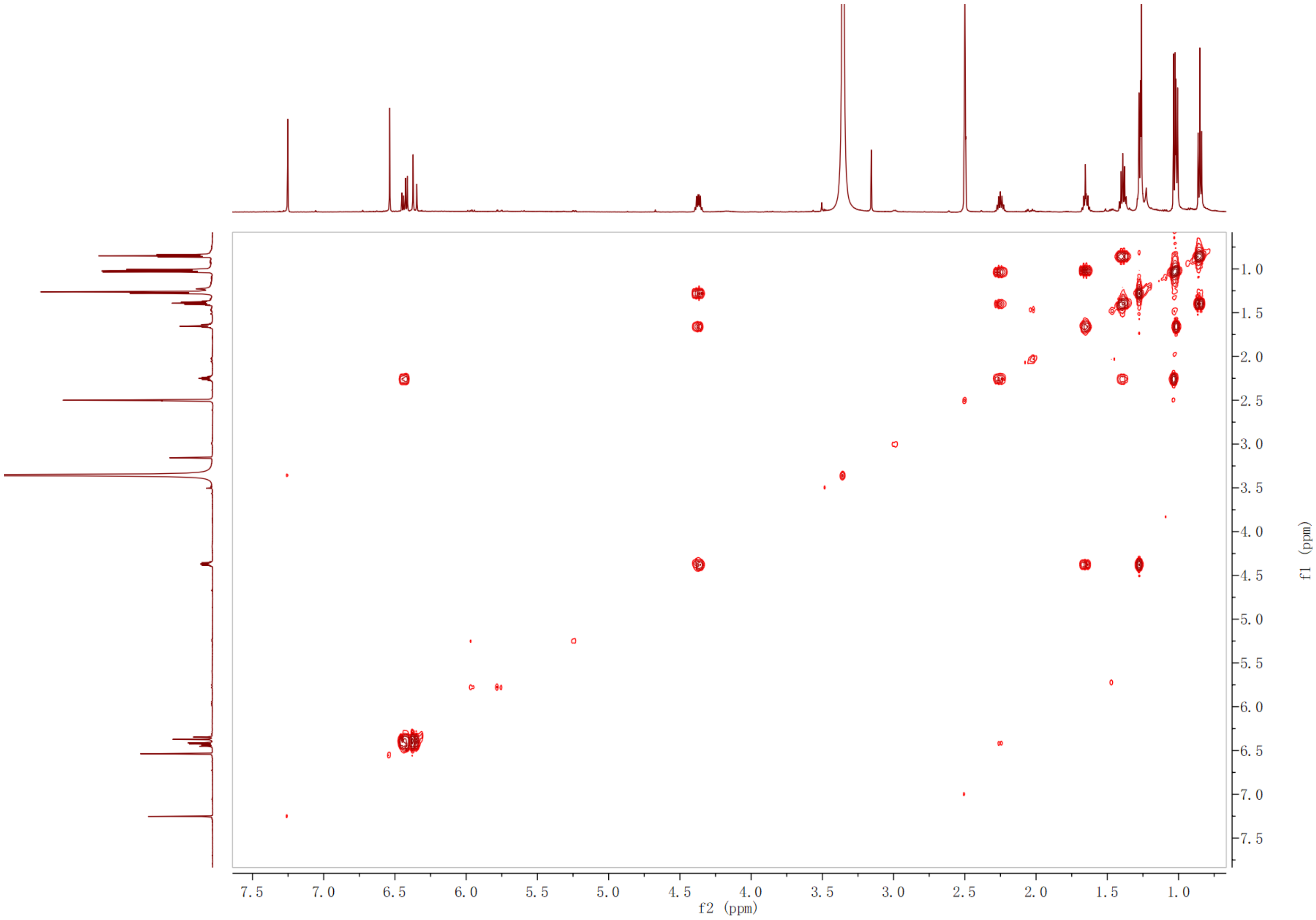

Figure S13. HMBC spectrum of chaetofanixin B (**2**) in DMSO-d_6

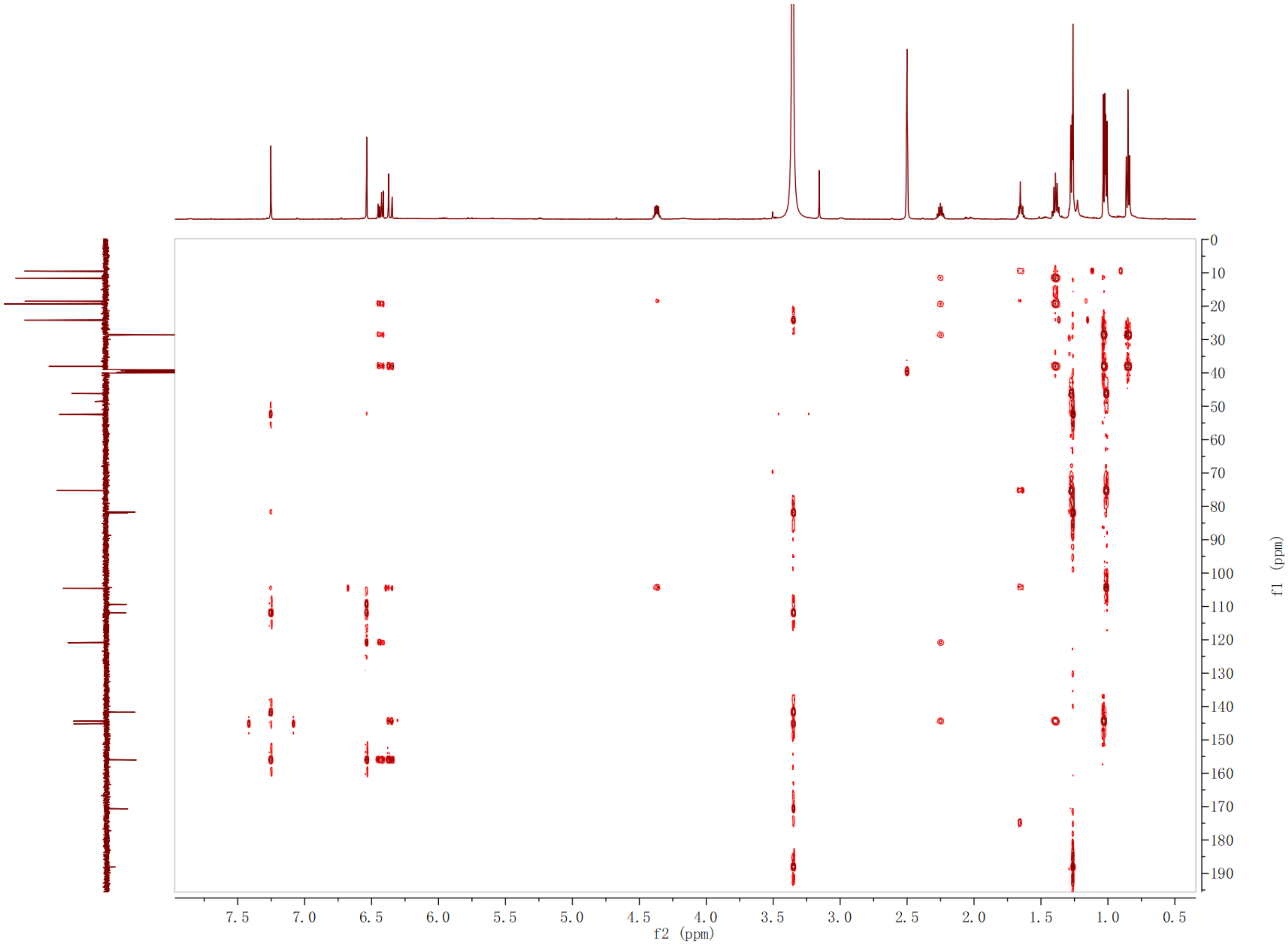

Figure S14. NOEZY spectrum of chaetofanixin B (**2**) in DMSO-d_6

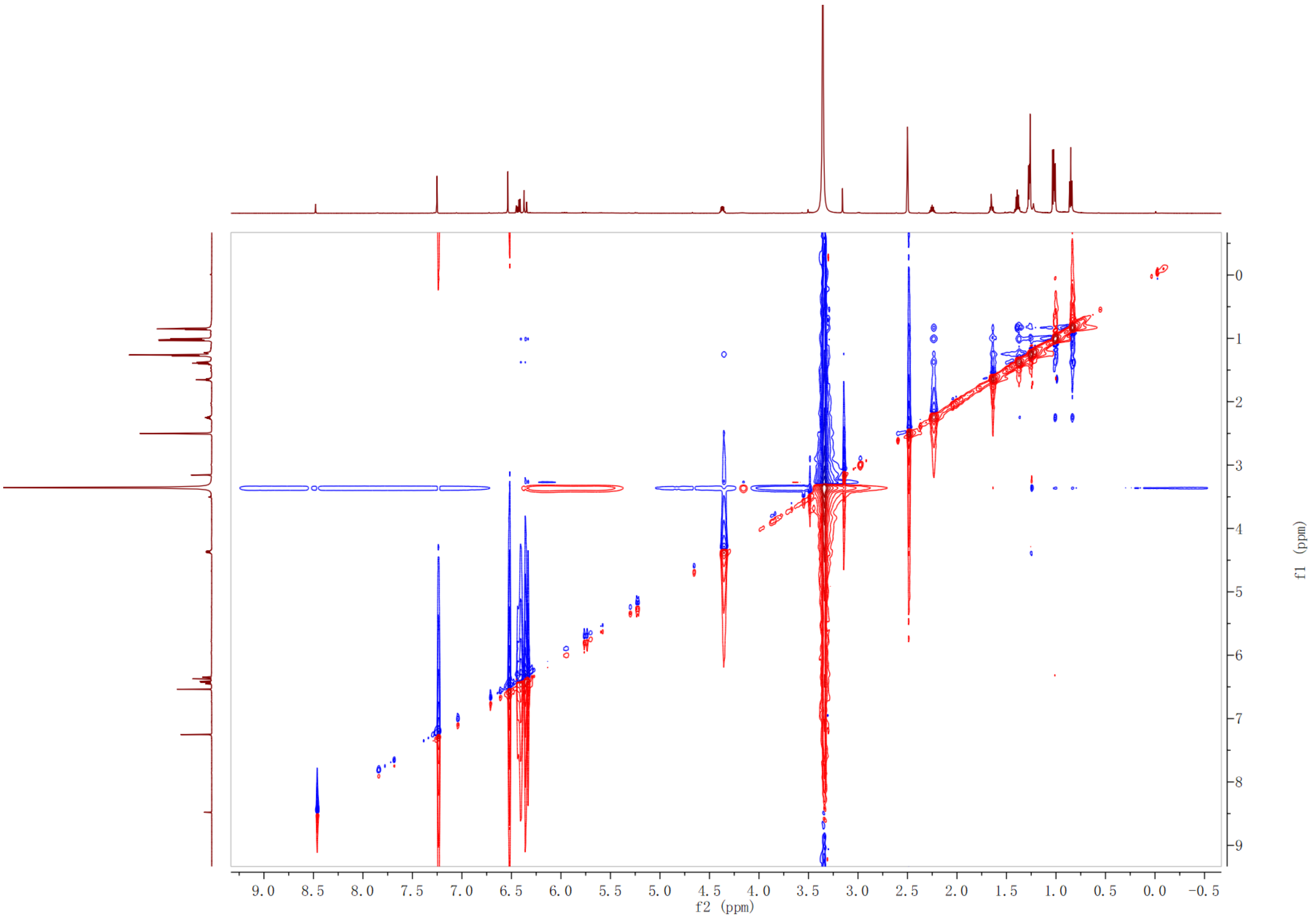

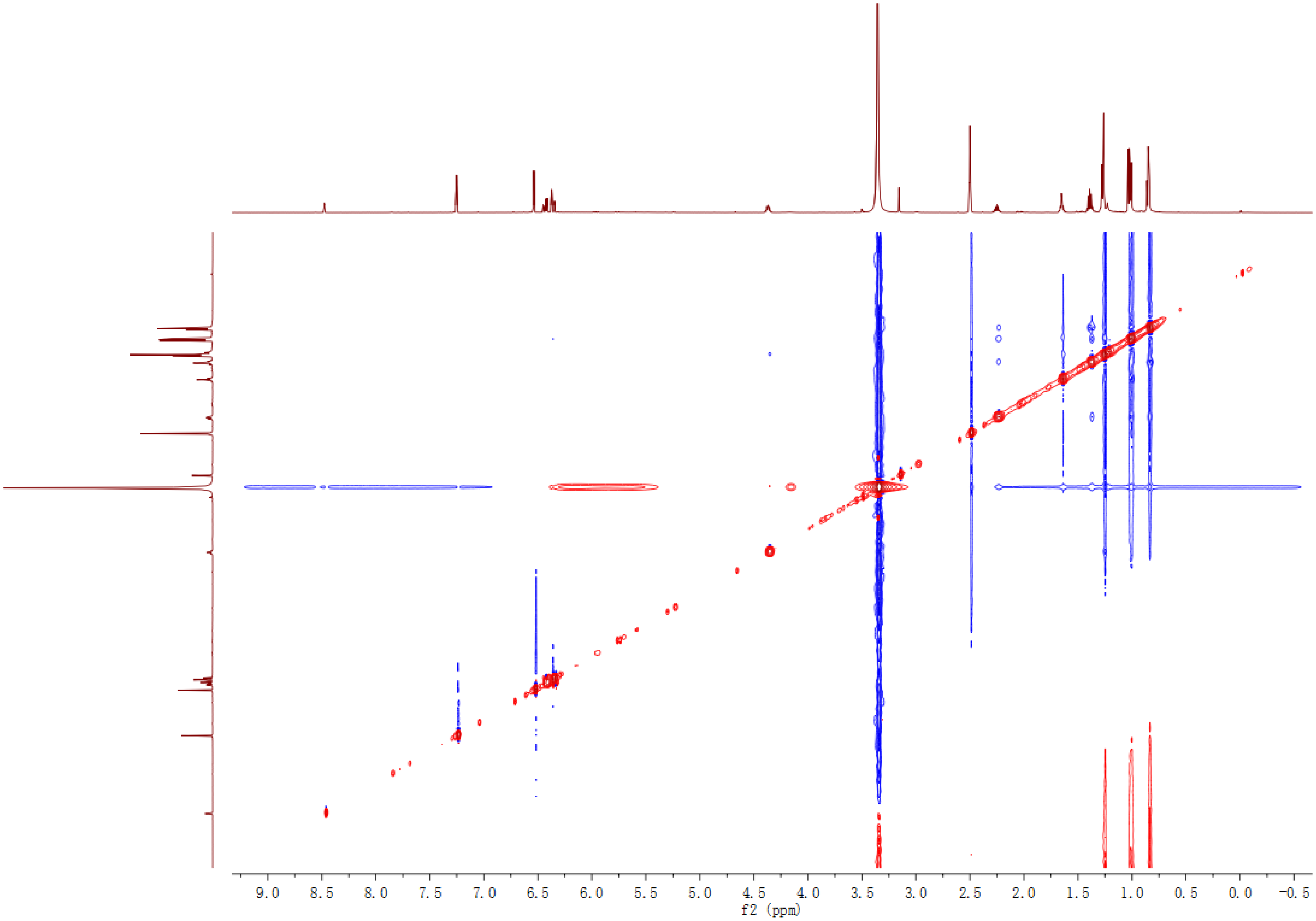
9.0
8.5
8.0
7.5
7.0
6.5
6.0
5.5
5.0
4.5
4.0
3.5
3.0
2.5
2.0
1.5
1.0
0.5
0.0
-0.5
f2 (ppm)

Figure S15. HRESIMS spectrum of chaetofanixin C (**3**)

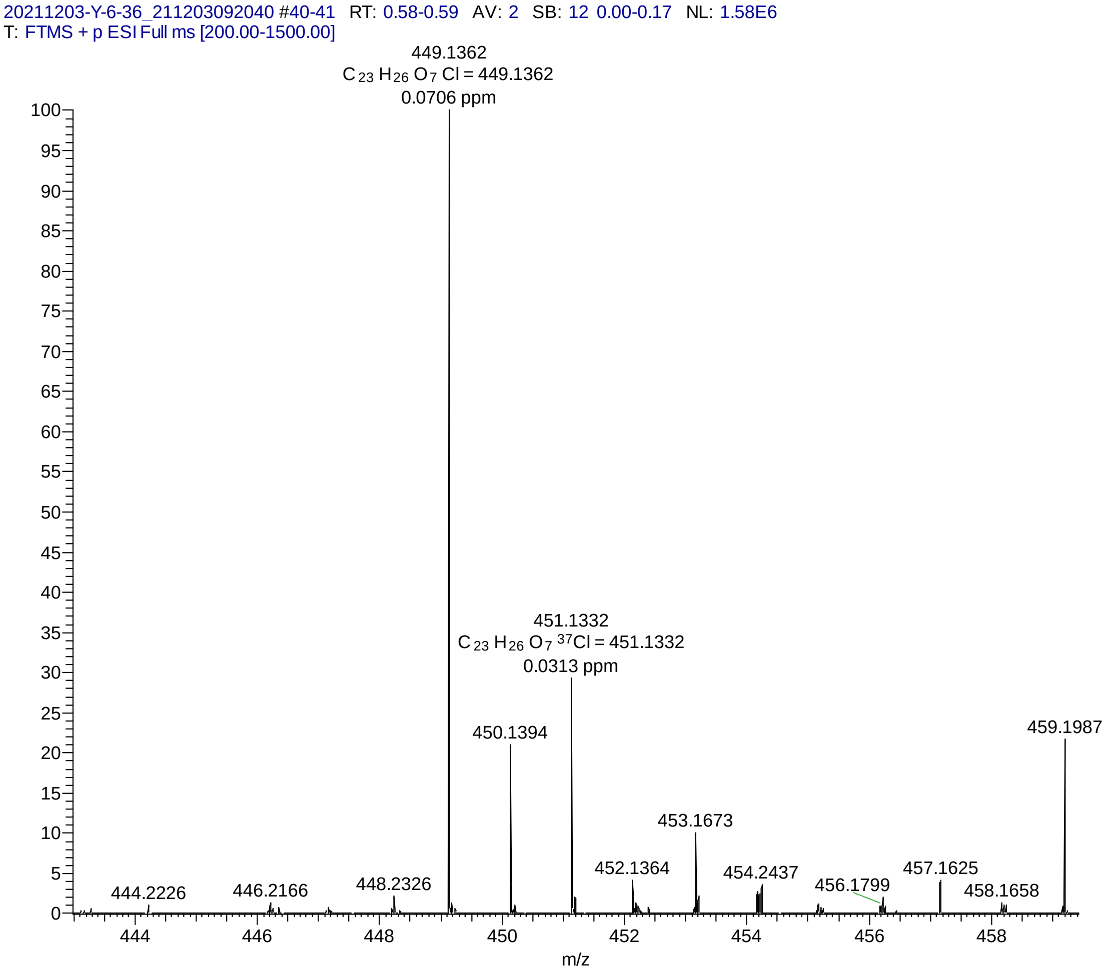

Figure S16. ^{1}H-NMR spectrum of chaetofanixin C (**3**) in DMSO-d_6

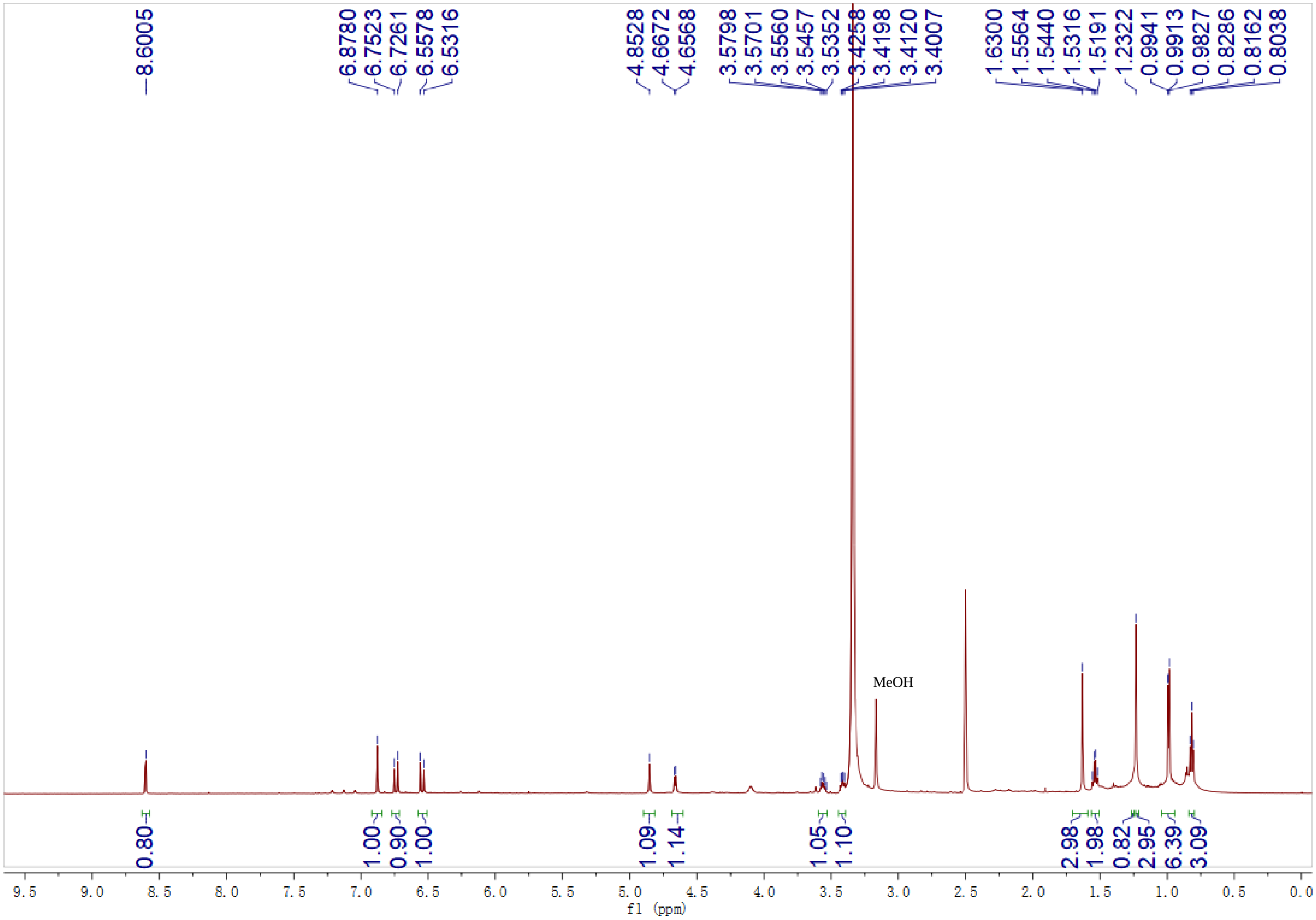

Figure S17. ^{13}C-DEPTQ spectrum of chaetofanixin C (**3**) in DMSO-d_6

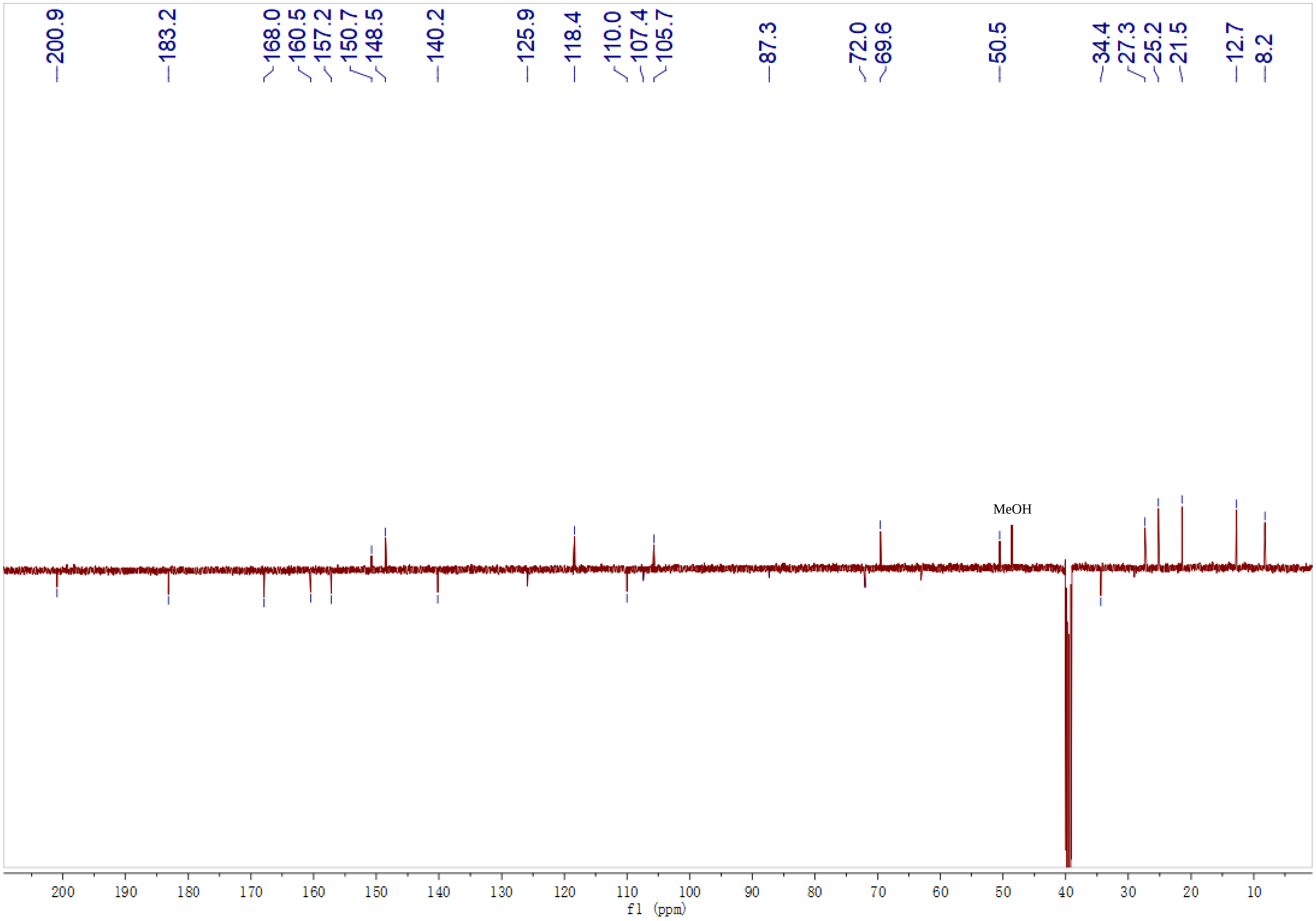

Figure S18. HSQC spectrum of chaetofanixin C (**3**) in DMSO-d_6

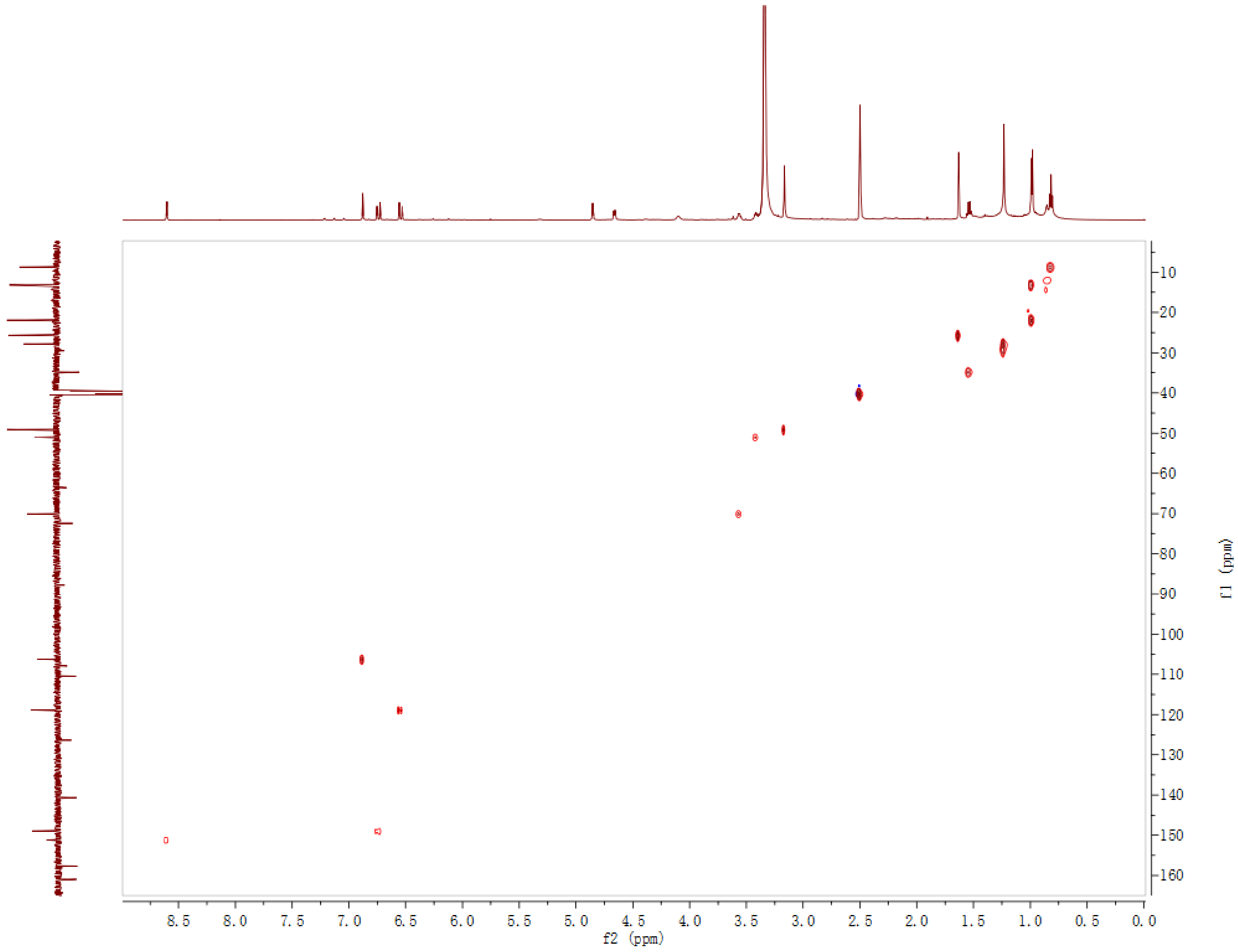

Figure S19. ^1H-^1H COSY spectrum of chaetofanixin C (**3**) in DMSO-d_6

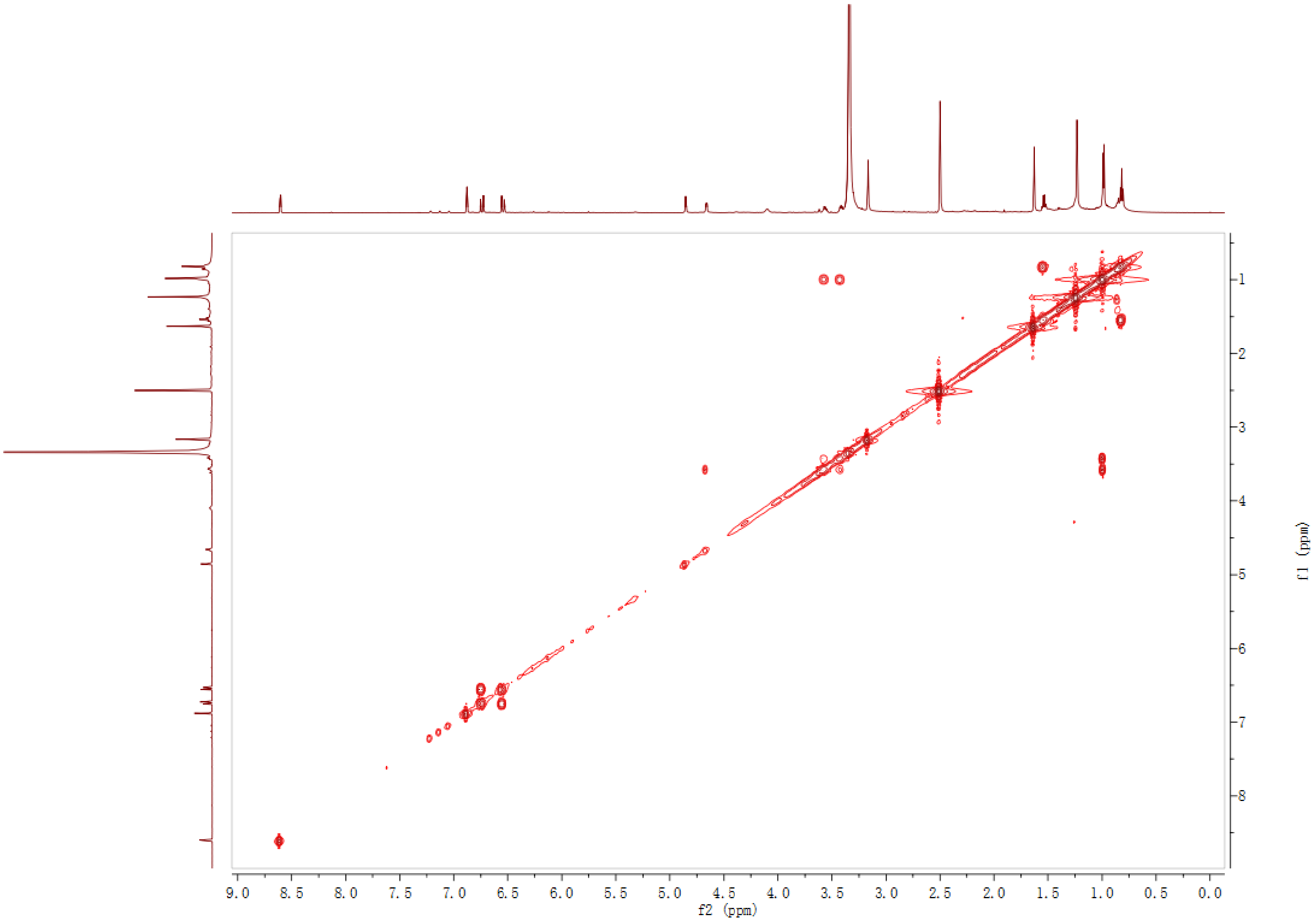

Figure S20. HMBC spectrum of chaetofanixin C (**3**) in DMSO-d_6

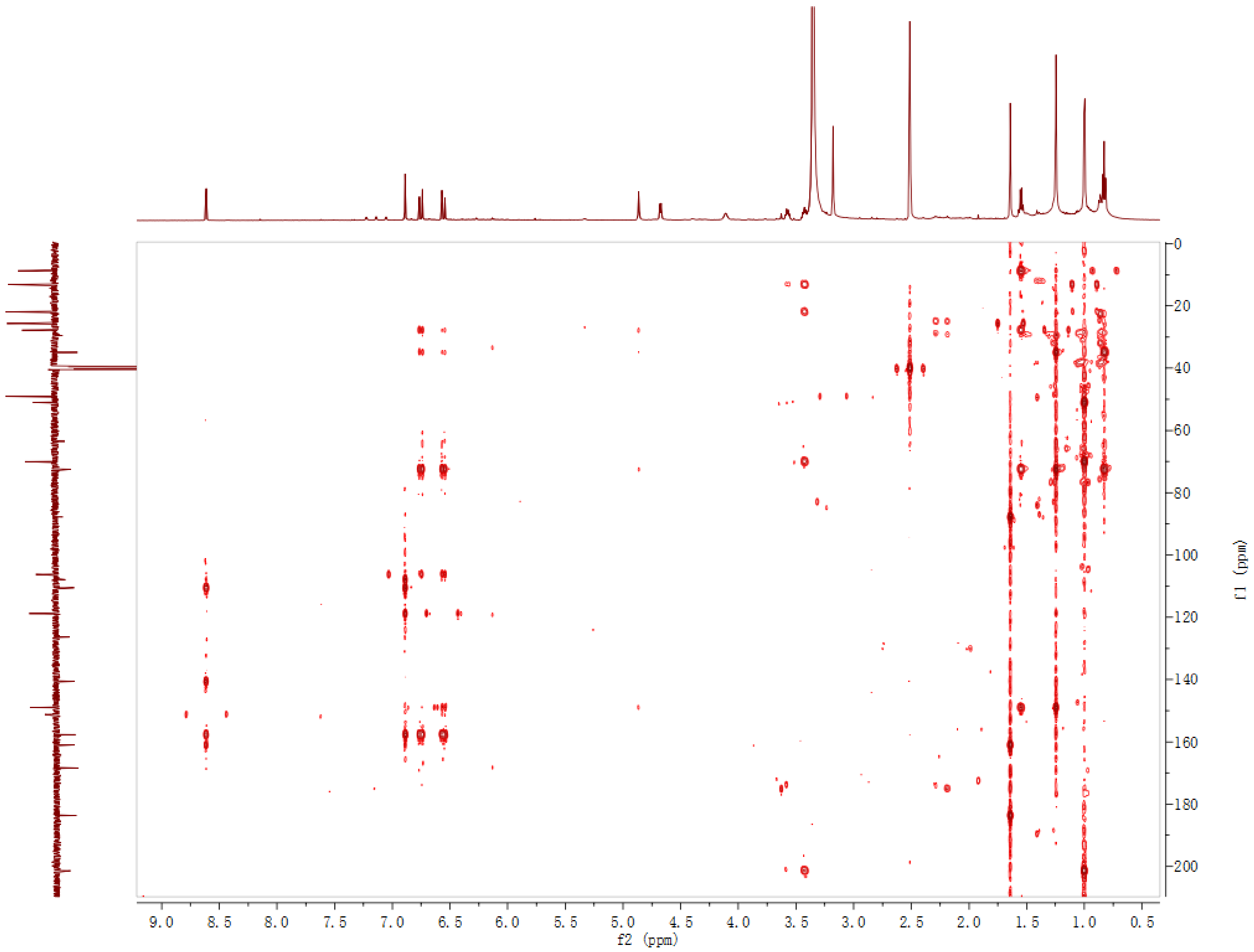

Figure S21. HRESIMS spectrum of chaetofanixin D (**4**)

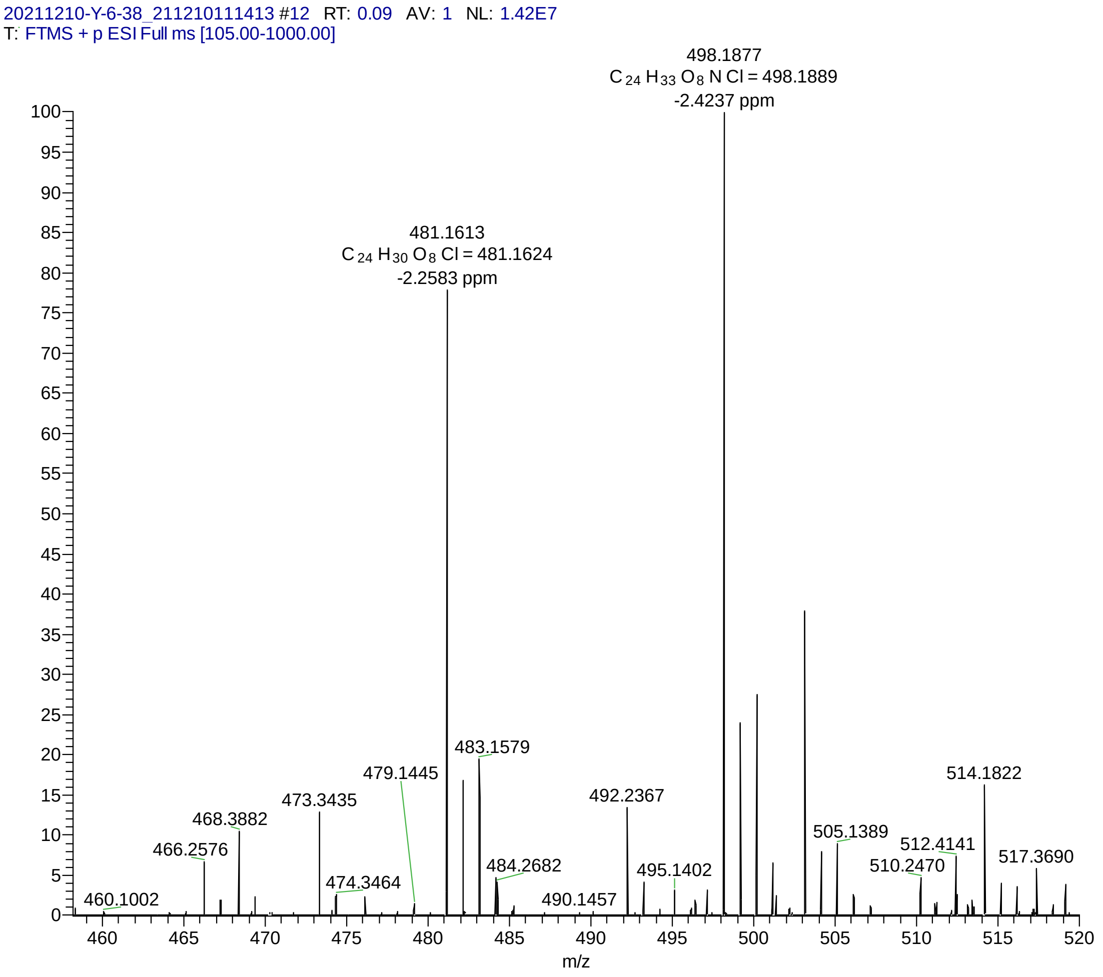

Figure S22. ^{1}H-NMR spectrum of chaetofanixin D (**4**) in DMSO-d_6

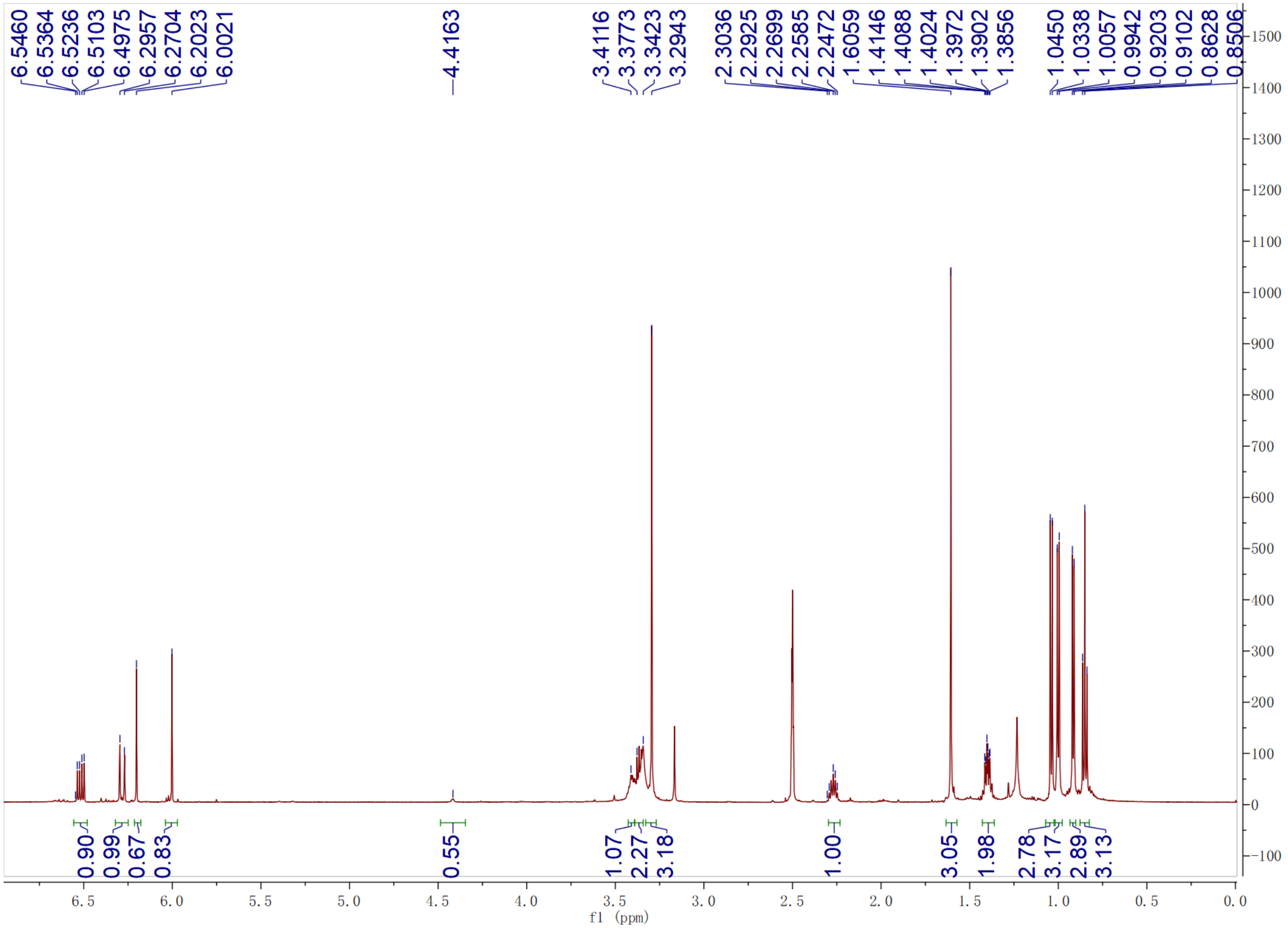

Figure S23. ^{13}C-DEPTQ spectrum of chaetofanixin D (**4**) in DMSO-d_6

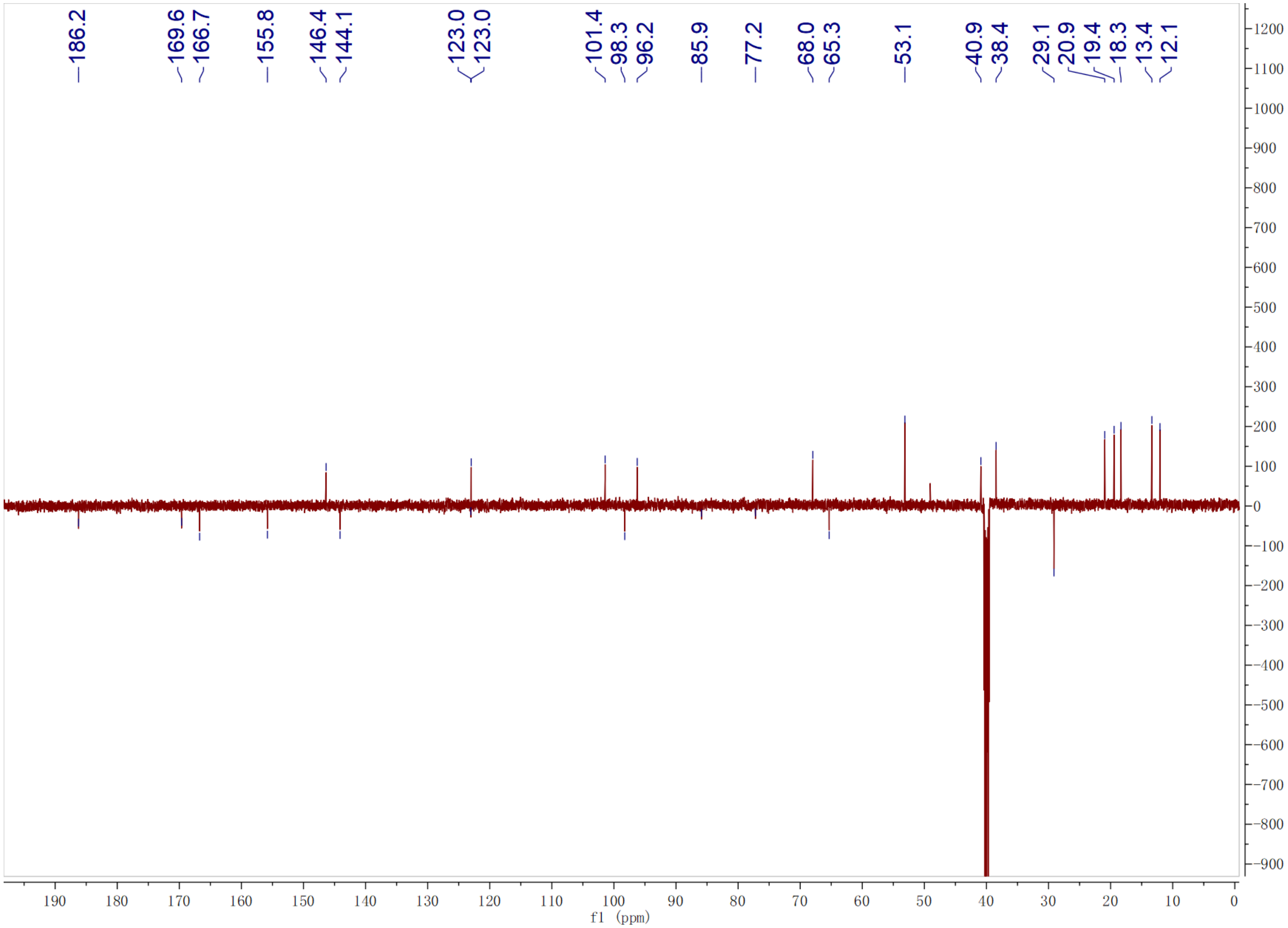

Figure S24. HSQC spectrum of chaetofanixin D (**4**) in DMSO-d_6

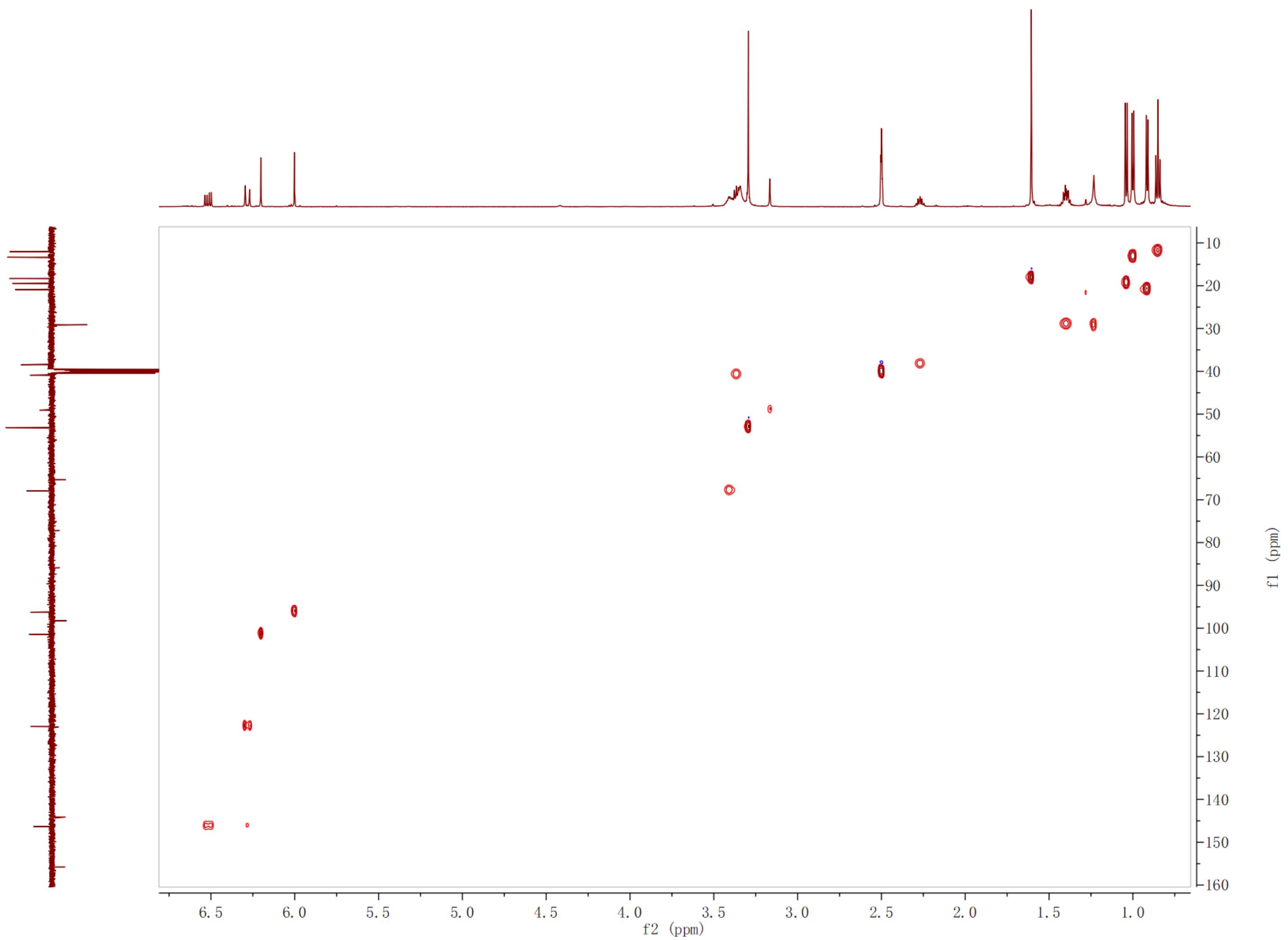
6.5
6.0
5.5
5.0
4.5
4.0
3.5
3.0
2.5
2.0
1.5
1.0
f2 (ppm)
10
20
30
40
50
60
70
80
90
100
110
120
130
140
150
160
f1 (ppm)

Figure S25. ^{1}H-^{1}H COSY spectrum of chaetofanixin D (**4**) in DMSO-d_6

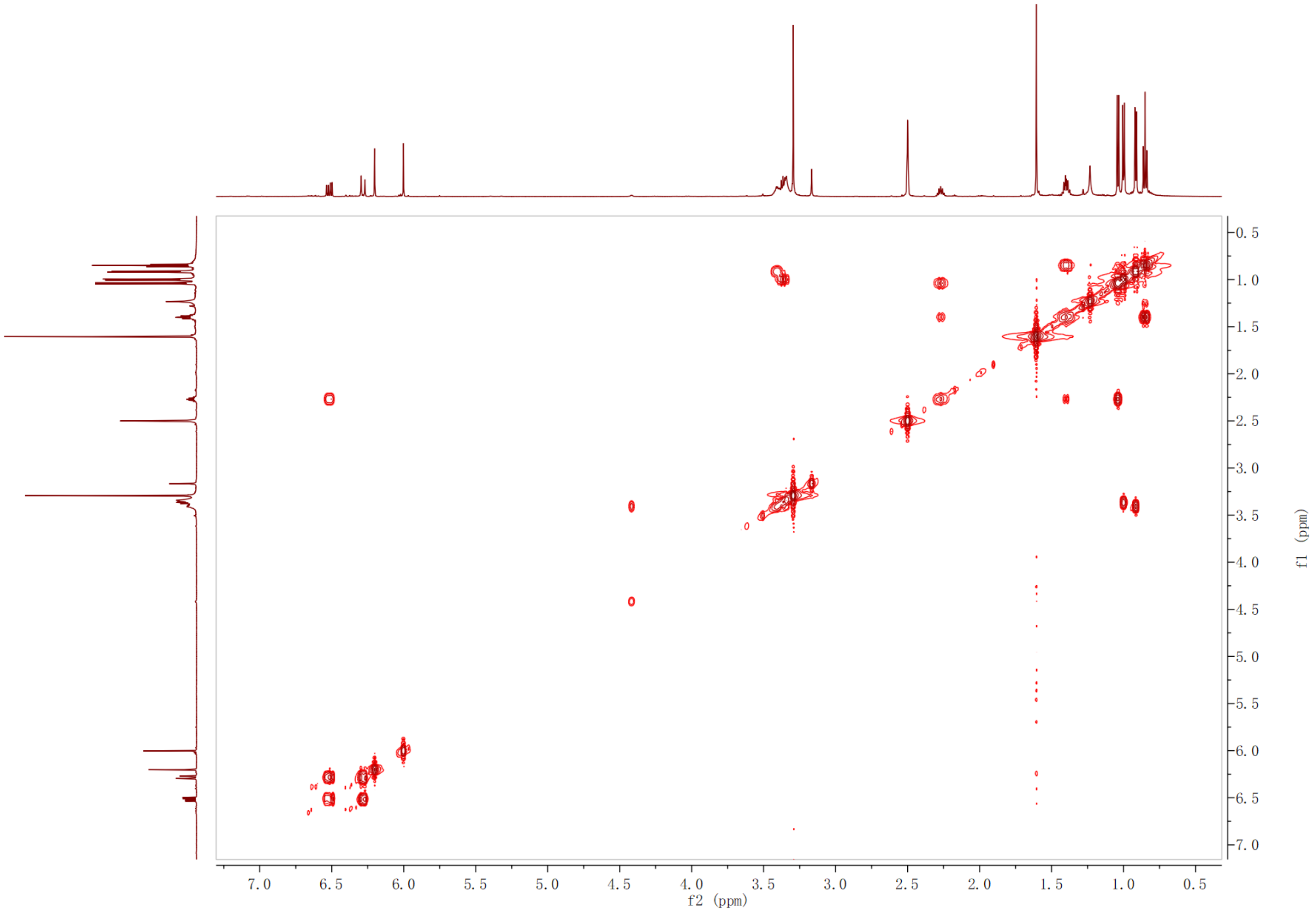

Figure S26. HMBC spectrum of chaetofanixin D (**4**) in DMSO-d_6

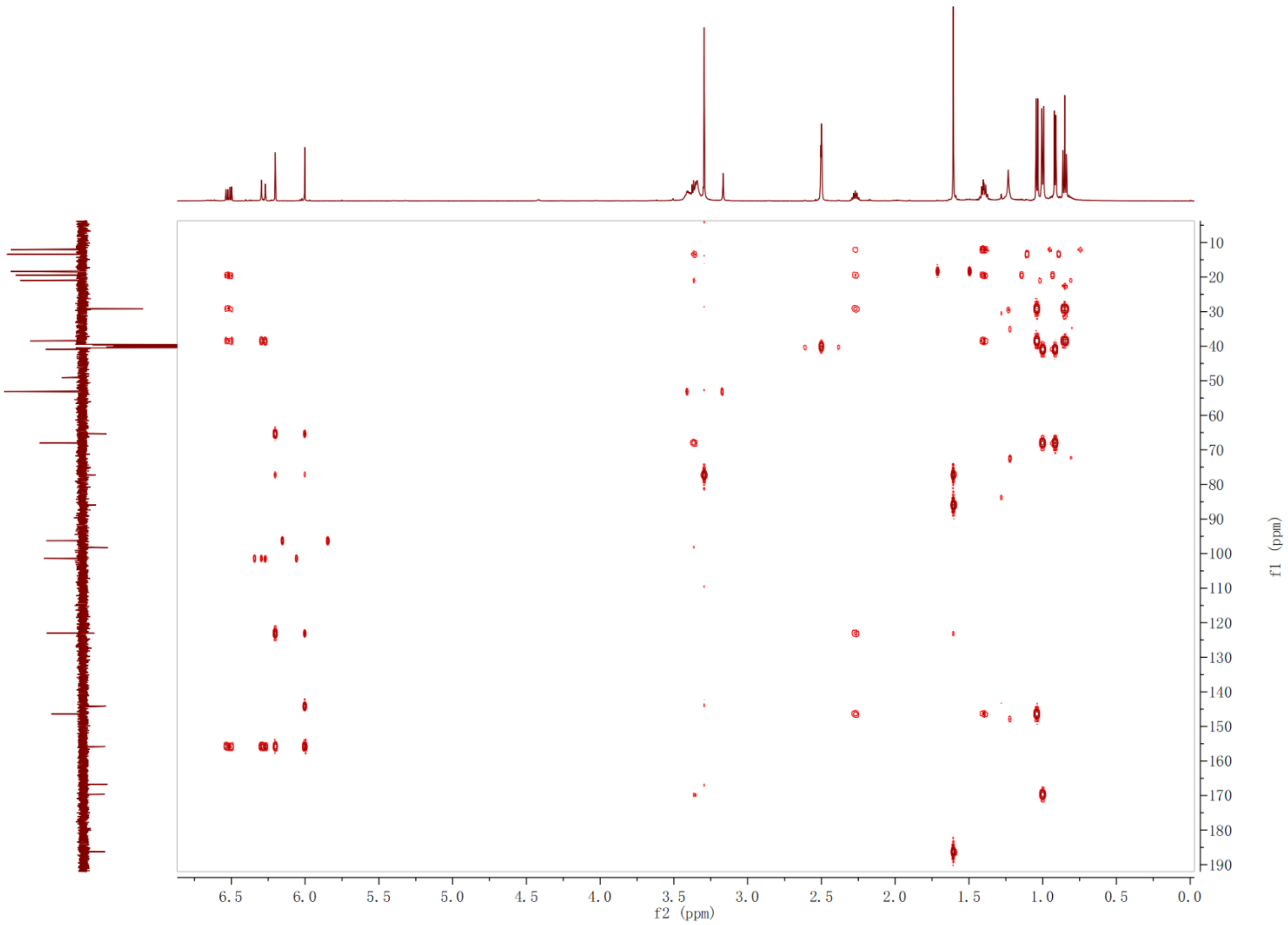

Figure S27. NOESY spectrum of chaetofanixin D (**4**) in DMSO-d_6

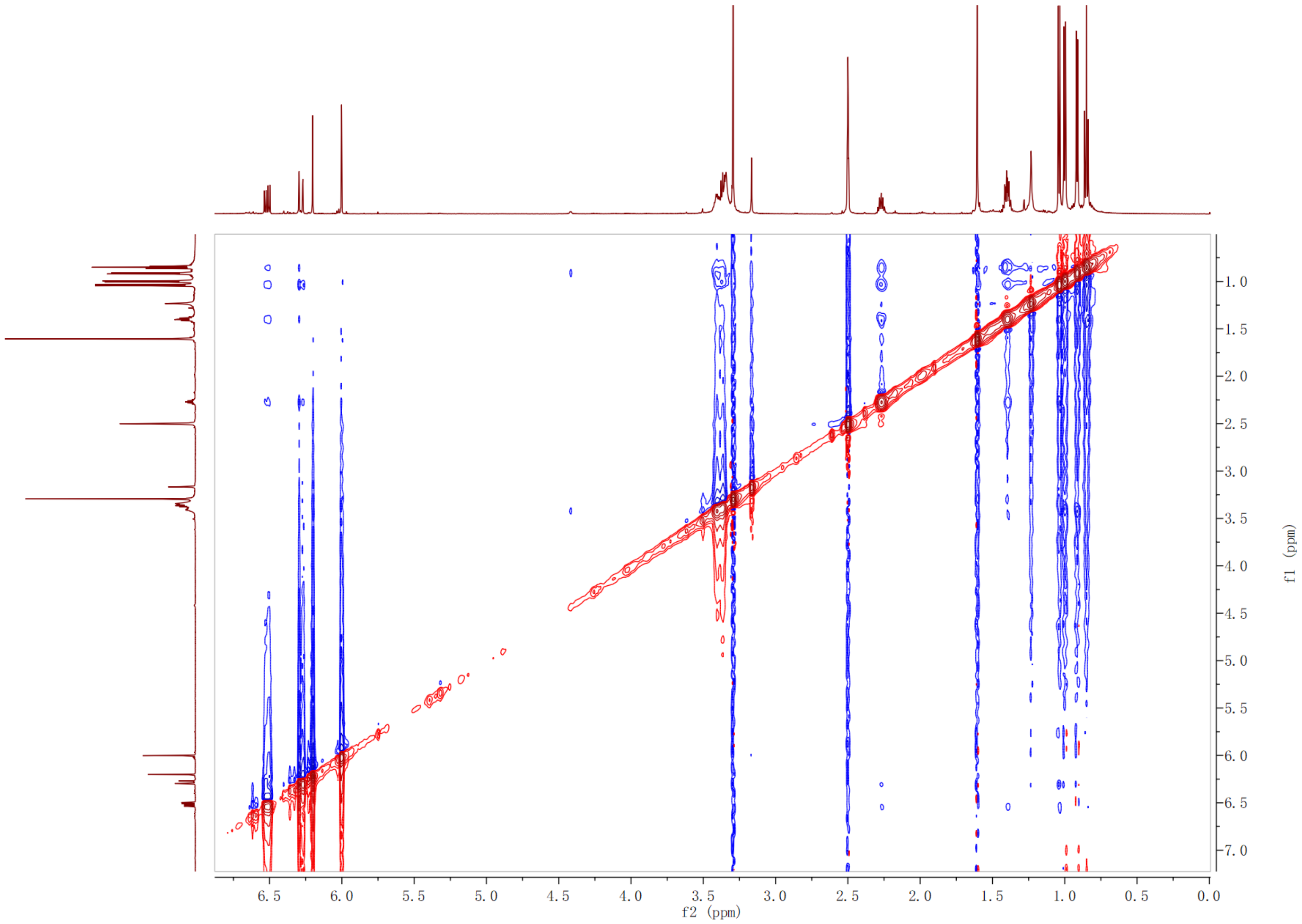

Figure S28. HRESIMS spectrum of chaetofanixin E (**5**)

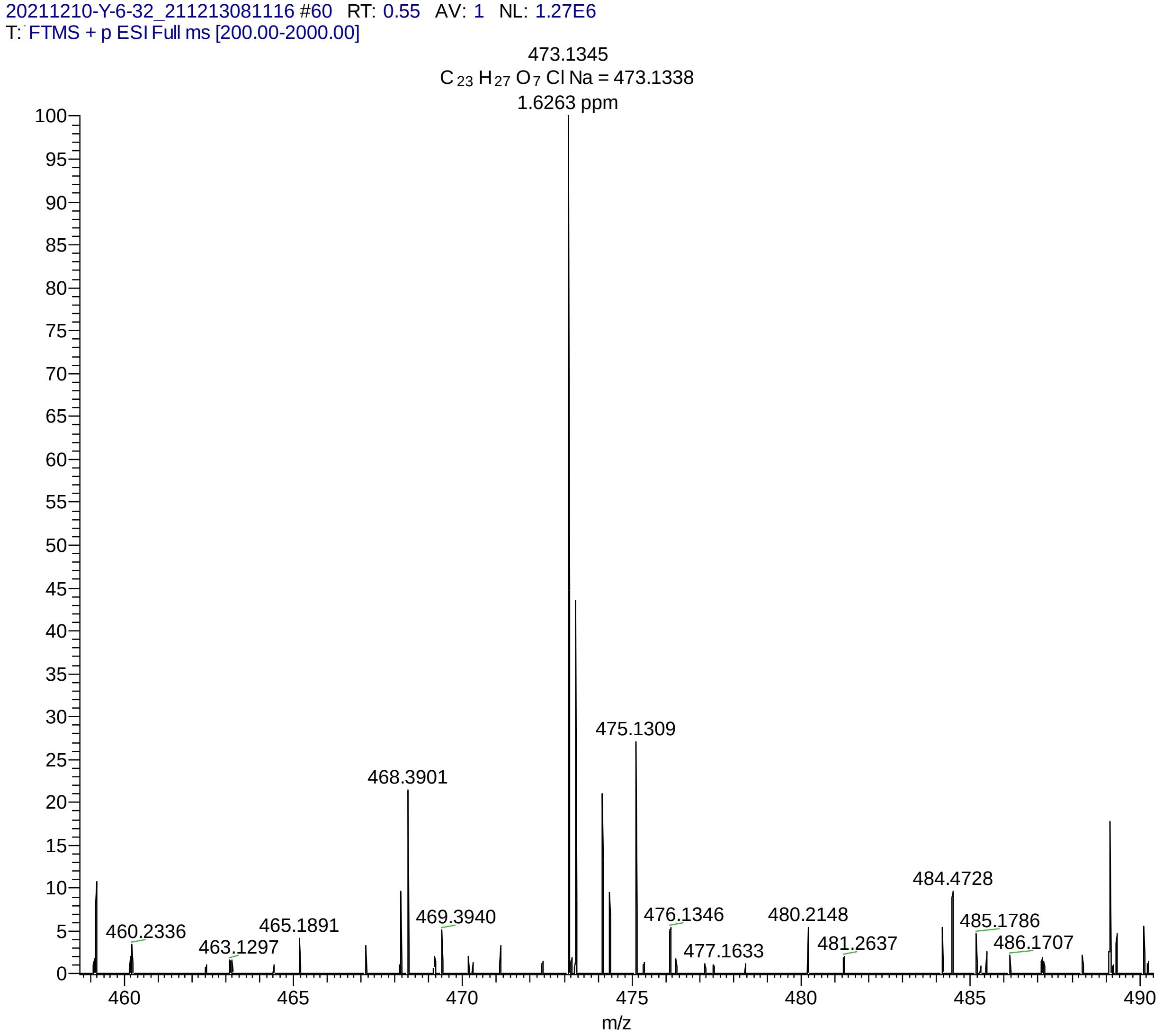

Figure S29. ^{1}H-NMR spectrum of chaetofanixin E (**5**) in DMSO-d_6

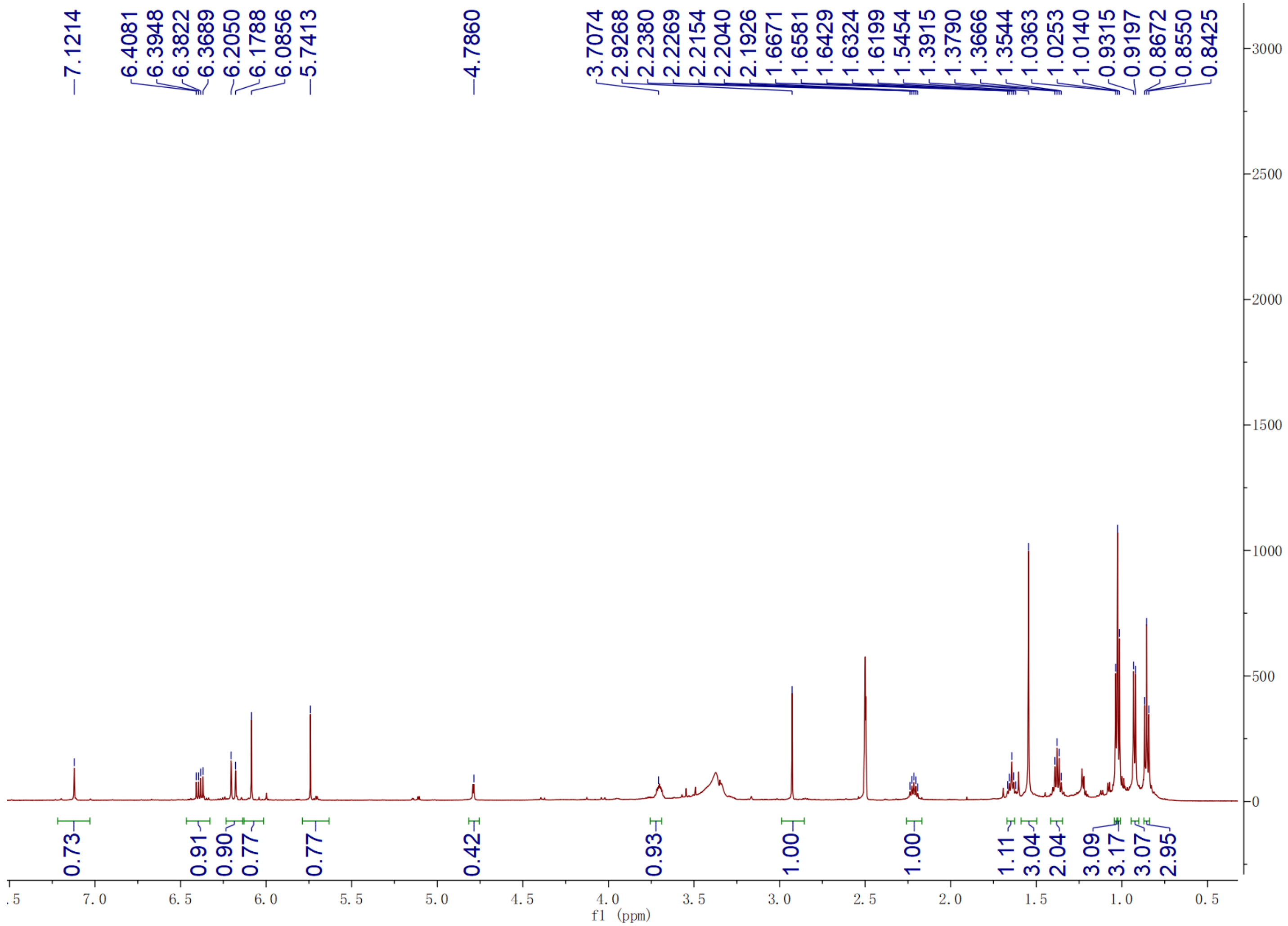

Figure S30. ^{13}C-DEPTQ spectrum of chaetofanixin E (**5**) in DMSO-d_6

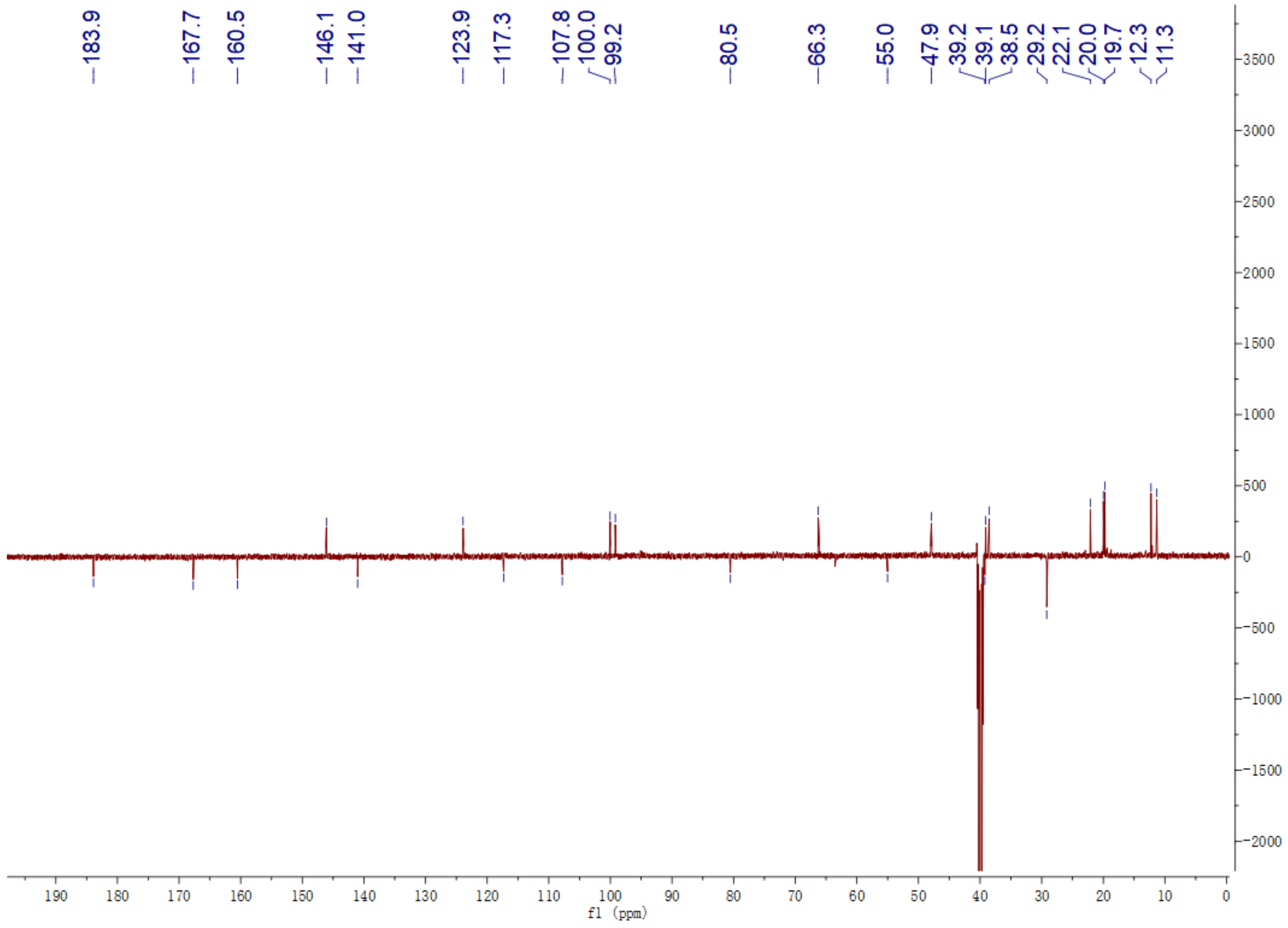

Figure S31. HSQC spectrum of chaetofanixin E (**5**) in DMSO-d_6

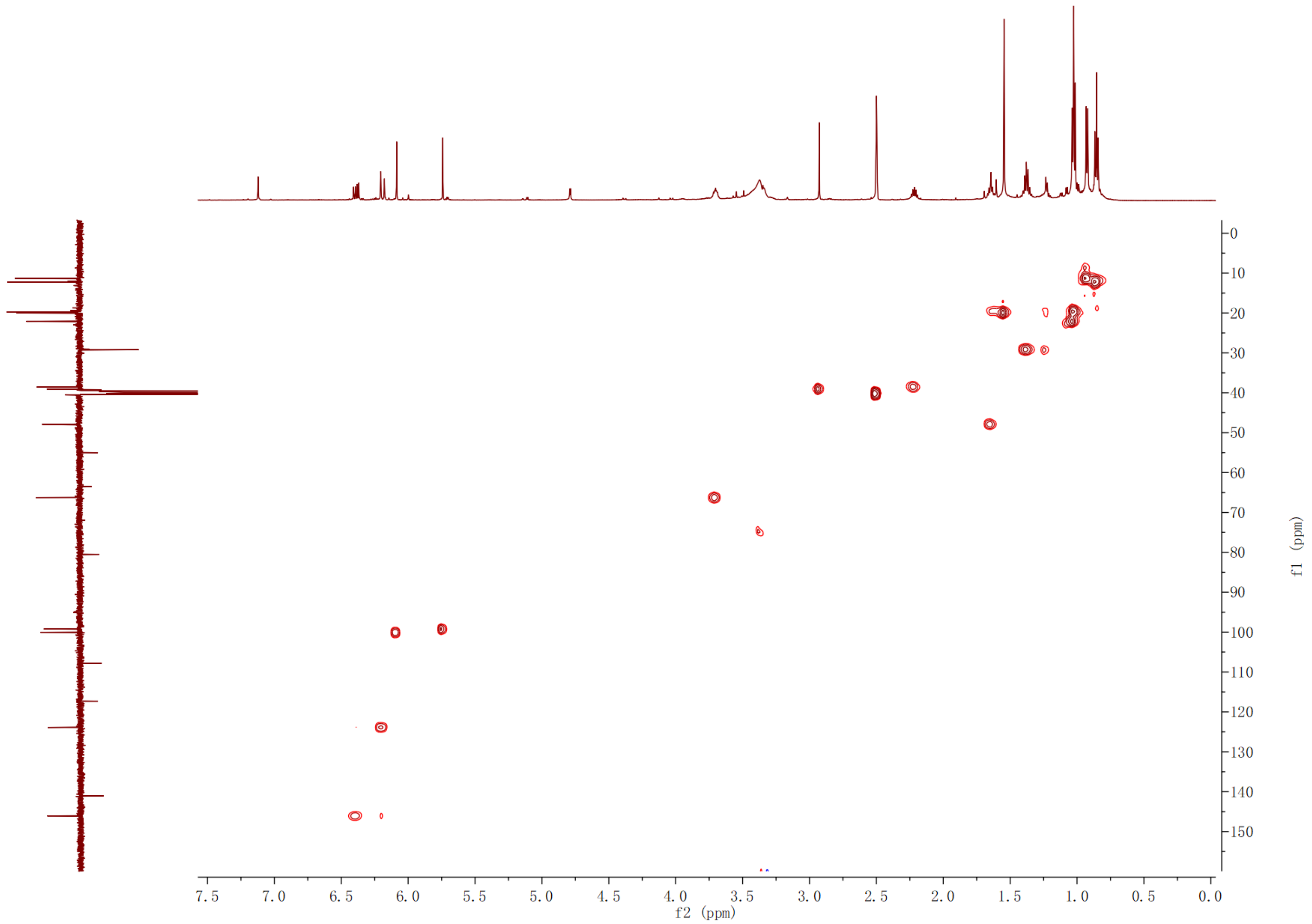

Figure S32. ^{1}H-^{1}H COSY spectrum of chaetofanixin E (**5**) in DMSO-d_6

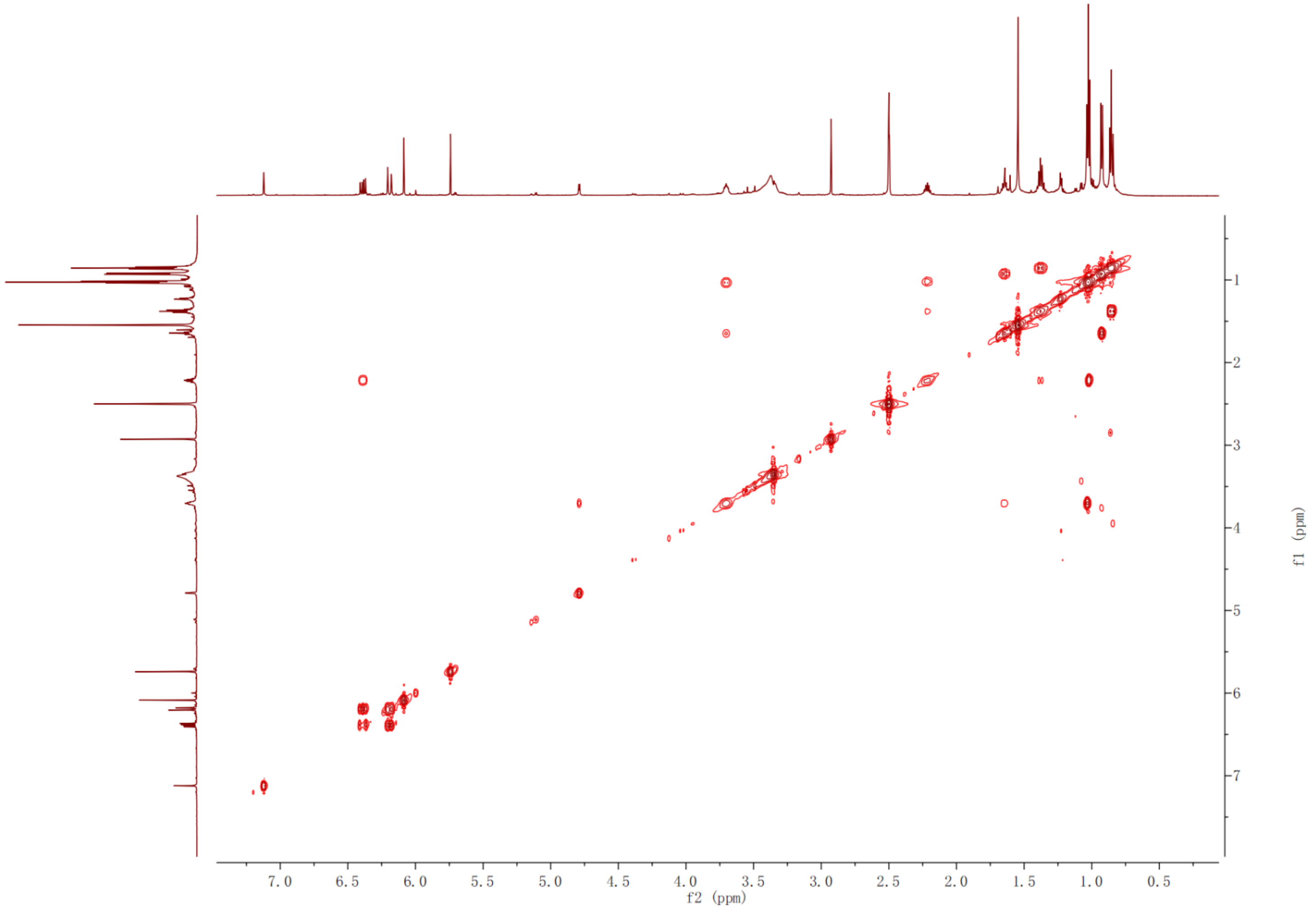

Figure S33. HMBC spectrum of chaetofanixin E (**5**) in DMSO-d_6

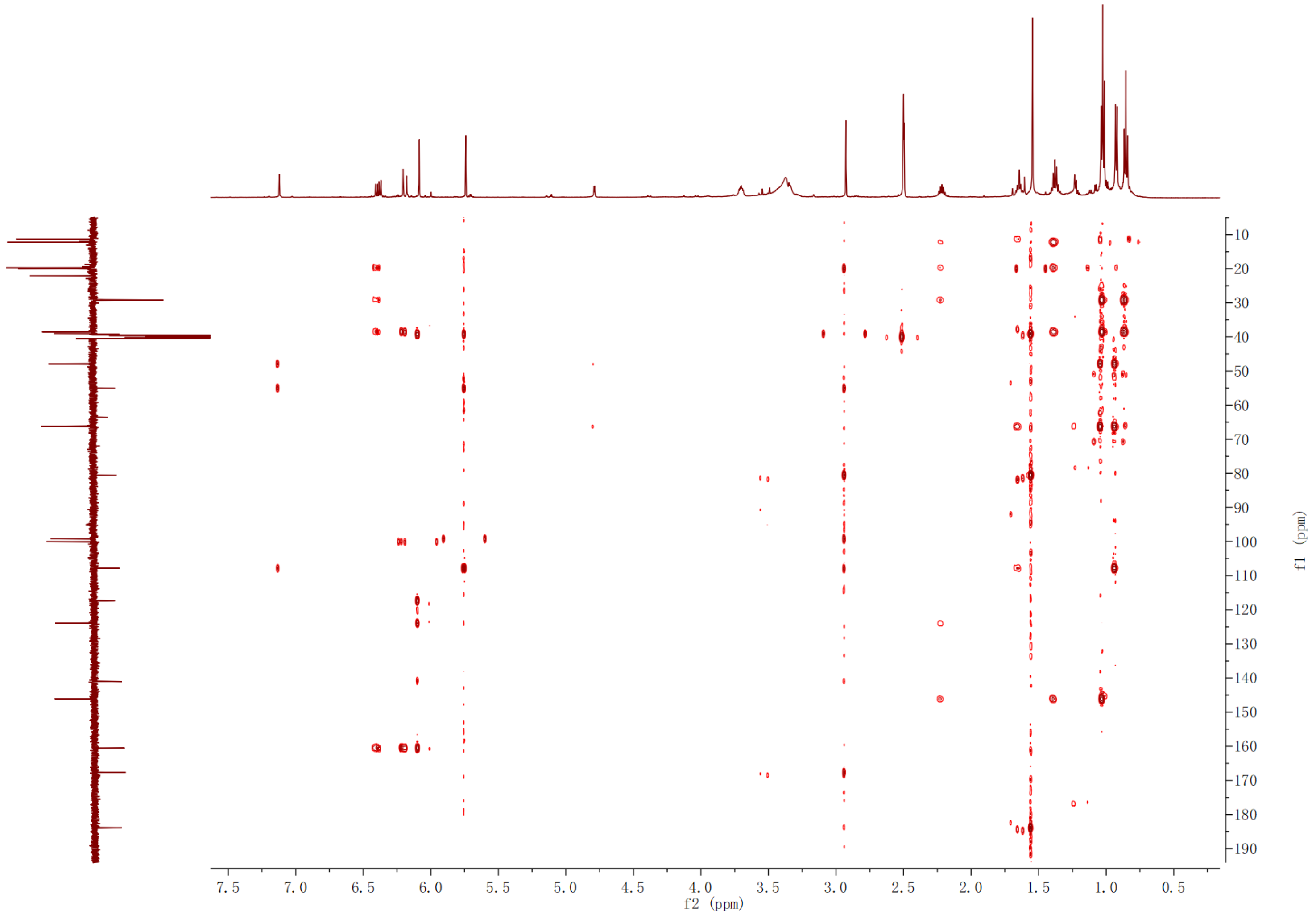

Figure S34. NOESY spectrum of chaetofanixin E (**5**) in DMSO-d_6

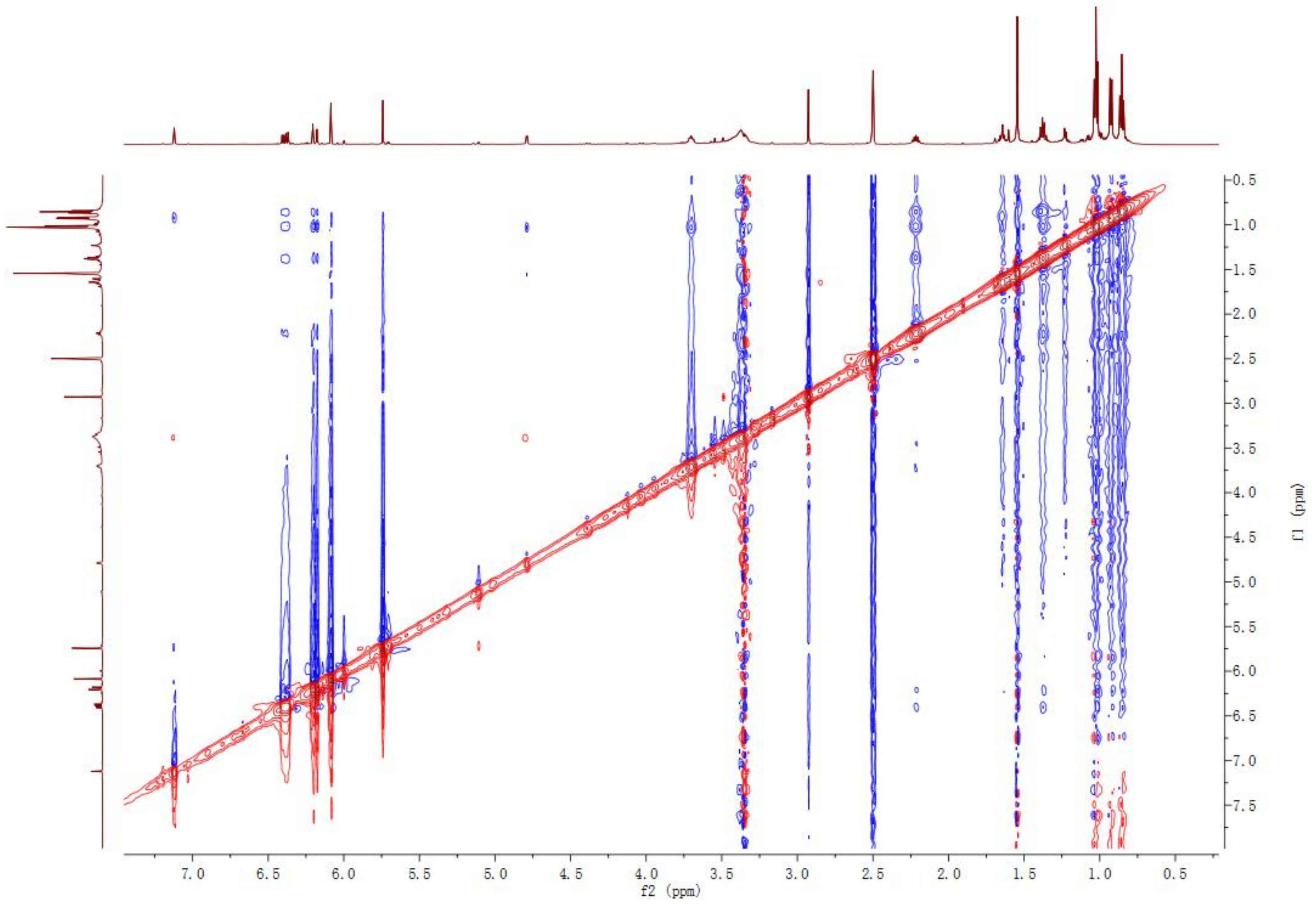

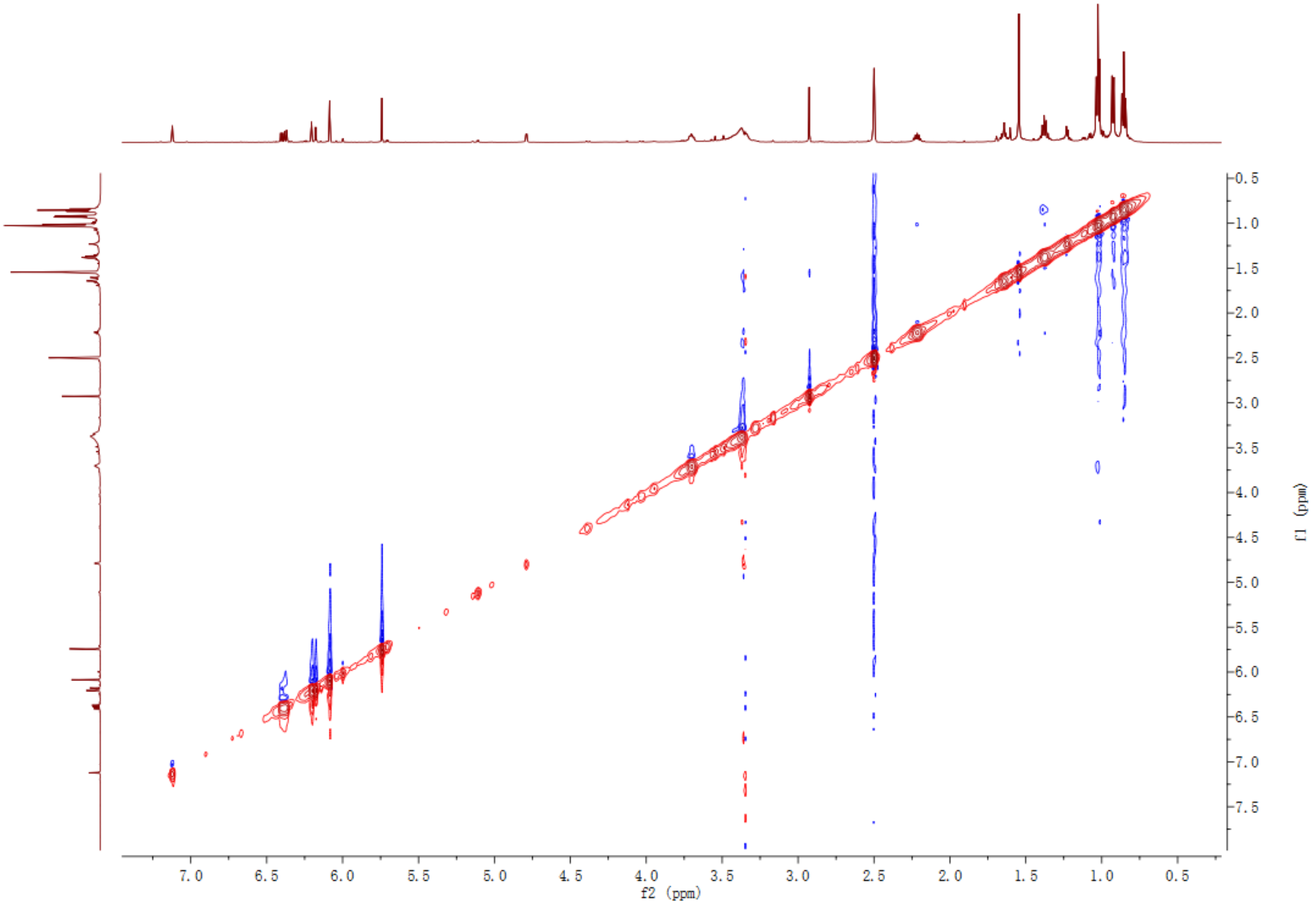
f2 (ppm)
f1 (ppm)
